# Genetic Consequences of Tree Planting Versus Natural Colonisation: Implications for Afforestation Programmes in the United Kingdom

**DOI:** 10.1111/eva.70146

**Published:** 2025-08-27

**Authors:** Guillermo Friis, Nicola Cotterill, Nadia Barsoum, Marcia Webberley, Mohammad Vatanparast, Michael Charters, Rômulo Carleial, Richard Buggs, James S. Borrell

**Affiliations:** ^1^ Royal Botanic Gardens, Kew Richmond Surrey UK; ^2^ Forest Research, Alice Holt Lodge Farnham Surrey UK

**Keywords:** afforestation, forest resilience, genetic diversity, genetic homogenisation, natural colonisation, pedunculate oak, planted woodlands, silver birch

## Abstract

The United Kingdom aims to dramatically accelerate the establishment of new woodlands by 2050, yet the impact of different afforestation strategies on landscape genetic diversity and resilience remains unclear. This study integrates environmental data, whole‐genome sequencing and phenotypic assessments to compare bioclimatic envelopes, genetic diversity and plant health indicators in naturally colonised versus planted populations of pedunculate oak and silver birch. We found that registered seed stands significantly under‐represent the wild bioclimatic envelopes of both species, as well as those of 21 out of 39 UK native species assessed, potentially limiting adaptive diversity in planted populations. Yet, genetic diversity metrics (*π*, *H*_O_ and *A*_R_) based on genome‐wide markers in planted populations were comparable to naturally colonised woodlands. Planted populations exhibited higher within‐group coancestry and moderate genetic homogenisation among sites, possibly reducing adaptive differentiation. Naturally colonised populations showed higher inbreeding coefficients (*F*_ROH_) in both species, potentially due to fragmentation of source populations. Genotype–environment associations based on redundancy analysis revealed divergent selection at functionally relevant loci, indicating distinct selective pressures in commercial tree production versus natural colonisation. Health indicators revealed reduced browsing in planted trees, and differences in mildew and leaf‐spot incidence, suggesting potential selection divergence between afforestation strategies. These findings support a role for both afforestation strategies in enhancing the resilience of future woodlands while highlighting pathway‐specific risks of introducing unintended impacts on forest diversity.

## 1 Introduction

Afforestation projects increasingly consider genetic diversity as integral to delivering resilient future tree populations (Aavik et al. 2012; Espeland et al. 2017; Jalonen et al. 2018; Mijangos et al. 2015; Shaw et al. 2025; Thomas et al. 2014). Consequently, the effects of alternative active and passive afforestation practices on population and landscape genetic diversity are the subject of growing attention (Jordan et al. 2019; Mijangos et al. 2015; Wei et al. 2023). The UK‐wide ambition to establish nearly a million hectares of new woodland by 2050 will be achieved through both active planting of trees raised from seed in nurseries and through passive natural colonisation in farmland, parkland and other open spaces (Forestry Commission 2022). Maintaining genetic diversity in newly established woodlands is crucial to their future resilience and a core objective of the UK Forest Genetic Resources (UKFGR) strategy (Trivedi et al. 2019). However, large‐scale afforestation efforts carry varying risks for preserving genetic diversity compared to wild populations. Here, we assess the impacts on contemporary population and landscape genetic diversity of alternative afforestation pipelines (encompassing both passive processes and human‐mediated decisions, from seed collection and nursery production to tree planting) for two widespread broadleaf tree species. By evaluating these contrasting strategies, we aim to clarify their potential contributions to the resilience of future woodlands and provide an evidence base to guide afforestation policy and practice in the United Kingdom.

**FIGURE 1:**
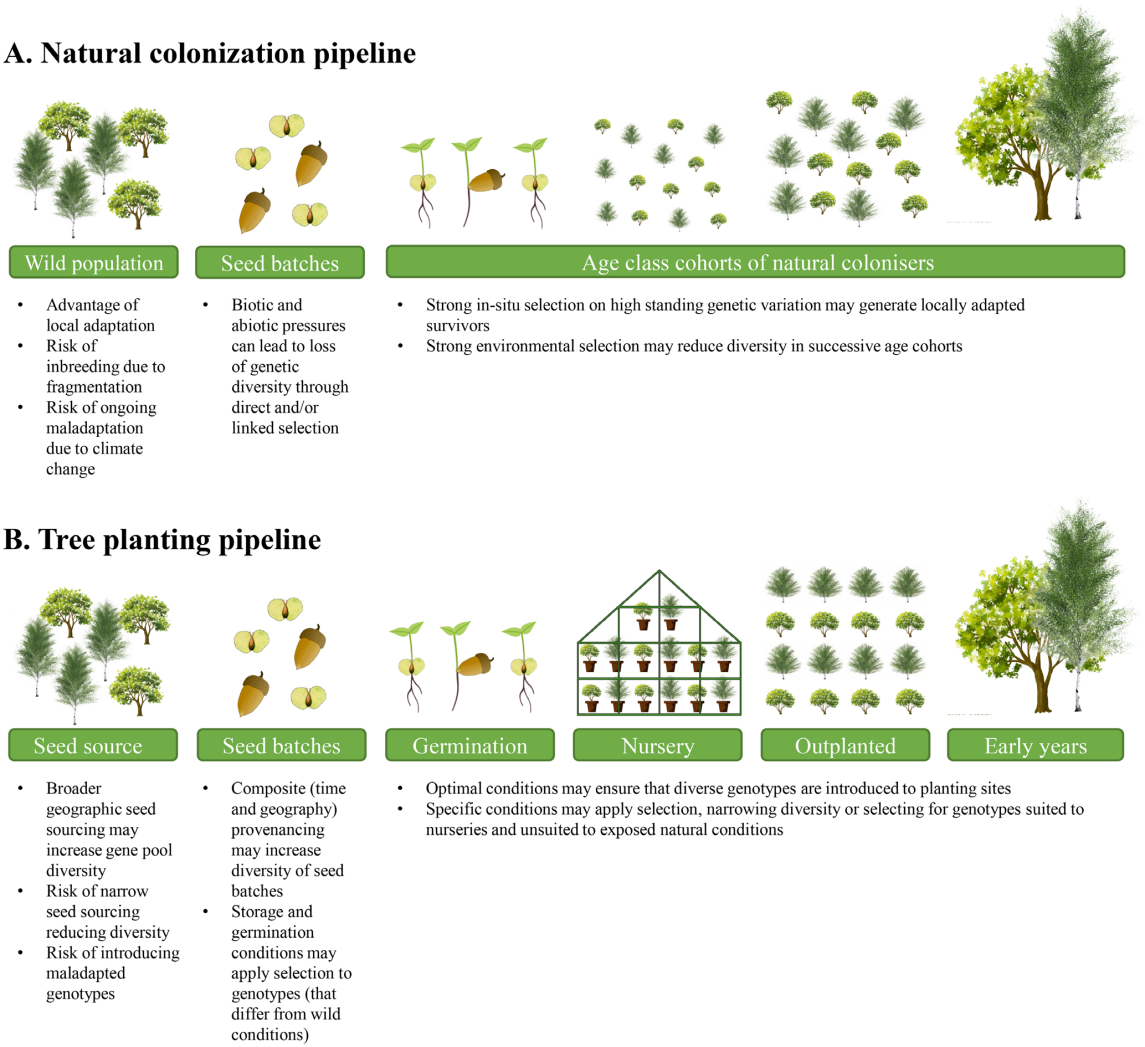
Comparative framework of factors with potential effects on genetic diversity patterns. Outlining of expectations for afforestation pipelines based on (A) natural colonisation and (B) active planting.

Afforestation strategies that rely on passive natural colonisation may benefit from broader genetic pools found in mature, parental stands. Resulting populations may be better adapted to the environmental conditions of target sites, facilitated by the presence of locally advantageous alleles within the local genetic pools or by natural selection acting on higher standing genetic variation during the various stages of establishment (Breed et al. 2018; Broadhurst et al. 2016). However, degraded landscapes are characterised by habitat fragmentation. Reproducing individuals confined to small, isolated patches can experience genetic depletion due to drift, and this can result in higher inbreeding of naturally established stands compared to planted sites of diverse origin (e.g., Campanella et al. 2013; Zavodna et al. 2015). Additionally, strong environmental pressures, pathogens, predation and other biotic stresses can reduce fecundity or recruitment, resulting in the loss of genetic diversity through both direct selection on functional loci and linked selection (Charlesworth et al. 1993; Espeland et al. 2017). Moreover, given the rapid pace of contemporary climate change, naturally colonised stands may originate from parental trees no longer suited to emerging conditions (Bradley St Clair and Howe 2007; Breed et al. 2018; Frank et al. 2017), resulting in locally maladapted gene pools and potentially limiting their adaptive capacity in altered environments (Figure 1A).

Conversely, afforestation through active tree‐planting pipelines risks introducing genetic bottlenecks at multiple stages, including seed collection, nursery cultivation and outplanting. The logistics of seed collection and industry demands for cost‐effective seedling production can limit the number of parent trees targeted and thus the genetic diversity that is introduced into planted populations (Jalonen et al. 2018; Jordan et al. 2019; Whittet et al. 2016). Although adverse effects can be minimised by following best‐practice seed collection protocols (Gosling 2007), they can still be particularly pronounced when local provenancing is prioritised and seed source trees occur in fragmented landscapes or small, isolated remnant populations with insufficient genetic diversity (Broadhurst et al. 2016). Additionally, limited availability of seed sources can lead to increased relatedness within seed stocks, heightening the risk of inbreeding at the population level and resulting in genetically homogeneous populations at the landscape scale. Conversely, the widespread use of uniform seed mixes across extensive areas may also contribute to genetic homogenisation, potentially increasing the likelihood of maladapted gene pools or outbreeding depression (Aavik et al. 2012; Breed et al. 2018; Höfner et al. 2022; Tong et al. 2020). In nurseries, standardised storage and germination conditions, along with protocols that favour reduced dormancy or visually appealing phenotypes during thinning—such as individuals with increased resource allocation to leaves—or that select for height during grading, may impose selective sweeps, further reducing genetic diversity (Espeland et al. 2017; Hoban et al. 2024; Wawrzyniak et al. 2020; Wei et al. 2023). The use of pesticides may also ameliorate selection pressures against trees that do not allocate resources to defence, resulting in higher frequencies of alleles that could be disadvantageous in natural environments. At the planting stage, failure to diversify sourcing from different nurseries or even different seed batches can further reduce genetic variation in newly established populations. As a result, cumulative bottlenecks may cause planted populations to diverge significantly from their original gene pool or from comparative wild populations (Espeland et al. 2017). Alternatively, nursery protocols that incorporate diverse provenances and prioritise high survival rates under optimal growth conditions may lead to more genetically diverse populations, potentially enhancing their capacity to adapt to emerging environmental challenges (Breed et al. 2013; Hoffmann et al. 2021; Jordan et al. 2019; Figure 1B).

Evaluating multiple indicators of genetic diversity in outplanted populations compared to natural stands provides an evolutionary perspective for assessing the success of restoration efforts (Jordan et al. 2019). In this study, we employ a multidisciplinary approach to address five policy‐relevant questions for UK afforestation programmes: (i) Do planting pipelines sufficiently represent the species' bioclimatic diversity?; (ii) Is genetic diversity in planting pipelines higher or lower than in naturally colonised populations?; (iii) Do planting pipelines alleviate inbreeding compared to naturally colonised populations?; (iv) Do planting pipelines generate populations with greater genetic homogeneity?; and (v) Do planting pipelines show evidence of selection?

To address these questions, we first compare the breadth of the climatic spaces or ‘bioclimatic envelopes’ (Pearson and Dawson 2003) of commercial tree seed sources and wild distributions for a large set of native UK species using gridded environmental data. We then focus on two native species—pedunculate oak ( *Quercus robur* ) and silver birch ( *Betula pendula* )—which have been intensively targeted for afforestation through both active planting and natural colonisation. Through extensive geographic sampling and whole‐genome sequencing, we assess patterns of genetic diversity and test for potential bottlenecks in planted versus naturally colonised woodlands across age cohorts of both species. We also examine whether selective pressures differ between recently outplanted populations and young natural colonisers using genotype–environment association (GEA) approaches. Finally, we evaluate differences in phenotypic health indicators between colonised and planted trees using generalised linear mixed models.

## 2 Materials and Methods

### 2.1 Bioclimatic Envelope of Commercial Tree Seed Sources Compared to Wild Distributions

To compare the bioclimatic envelopes of wild tree populations and commercially produced seed sources, we performed principal component analysis (PCA) on climatic data extracted from geographic locations and summarised the environmental space occupied by each group using minimum convex polygons. We started by collating a consensus list of 45 native UK tree species, based on data by the Forestry Commission (2019) and on Henniges et al. (2022). We excluded species with possible hybrid parentage ( *Salix fragilis* ), the genus *Populus* as very few of the registered stands listed the specific species, as well as most large shrub growth forms (Table S1). Occurrence data for the approximate wild distribution of species was obtained from the Plant Atlas 2020 (https://plantatlas2020.org/; Stroh et al. 2023).

Information on seed stand distributions was obtained from two different sources. First, we collated data from the UK Register of Basic Materials (UKRBM, https://data‐forestry.opendata.arcgis.com/datasets/, accessed 27 July 2023) which provided 328 points across 15 study species. Second, we collected data from Forest Reproductive Materials (FRM) master certificates provided by the Forestry Commission on seed harvests conducted in registered seed stands between 2015 and 2022. FRM master certificates record the number of collections per site, species (*n*), provenance region and basic elevation zone information (> 300 or < 300 m), yet not exact locations. Thereby, we used this information to generate surrogate seed collection location data points by randomly producing *n* points per site and species within the equivalent seed zone (Herbert et al. 1999) and elevation as registered in the certificates. In total, seed source data points sufficient for analysis (≥ 3) could be recovered for 39 of the original 45 native species in our list (Table S1).

Climate data were obtained from the Met Office at 1 km^2^ resolution for the entirety of the United Kingdom (Hollis et al. 2019). Data comprised annual means for 1991–2020 and consisted of 11 bioclimatic variables: total mm rainfall; average of daily mean, daily maximum and daily minimum air temperature; hours of bright sunshine; average of hourly mean wind speed at 10 m above ground level; average of hourly mean sea level pressure; average of hourly relative humidity; average of hourly vapour pressure; days with grass minimum temperature below 0°C; and days with over 50% snow cover at 0900 UTC. Climate information was extracted for each stand and surrogate seed collection location using the R (v4.2.2; R Core Team 2022) package ‘raster’ (Hijmans 2025).

To compute the bioclimatic envelopes, environmental data were scaled, and PCAs were run on the individual species datasets. PCs 1 and 2 were plotted, and the ‘vegan’ (Oksanen et al. 2016) function ‘ordihull’ was used to create minimum convex polygons of the source and wild sets of points, representing the bioclimatic range of each group. To test for significant differences, a PERMANOVA was run on each species based on the scaled set of climatic variables, using Euclidian distance. Polygon areas, polygon intersections and the percentages of the wild polygon intersected were also computed.

### 2.2 Study System and Sampling Design for Comparative Genomic Analyses

**FIGURE 2:**
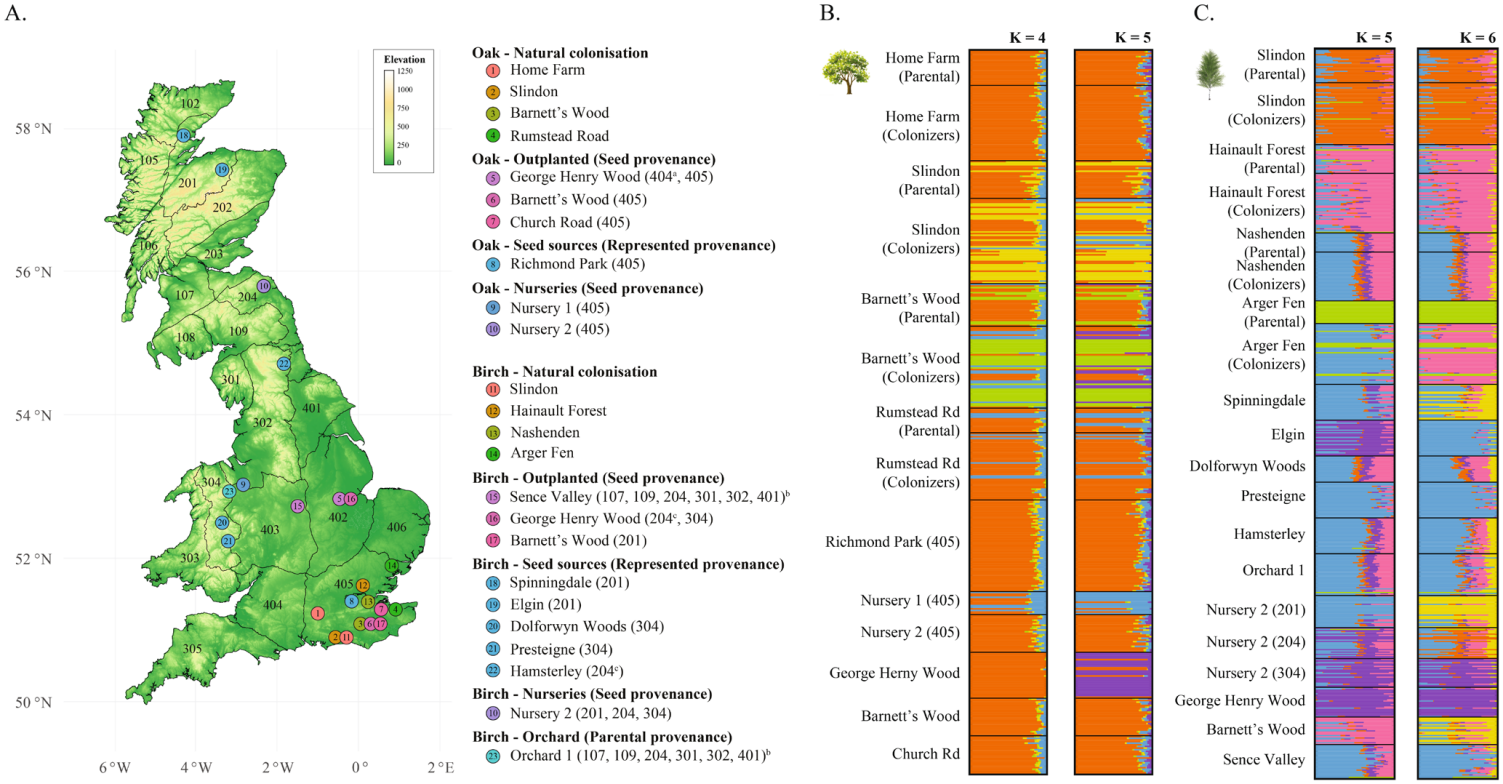
Geographic sampling and neutral population structure. (A) Sampling sites for pedunculate oak and silver birch, including wild natural colonisation sites, seed source representatives, nurseries, planted populations and the single orchard included in this study. The seed zone provenance of nursery and planted trees, as well as the represented seed zones of seed sources and the orchard, are indicated in parentheses. (B, C) Ancestry proportions estimated with ADMIXTURE for oak (*K* = [4, 5]) and birch (*K* = [5, 6]) respectively, based on 777,742 and 323,425 independent SNPs. Plots for lower values of *K* are reported in Figure S5. Colours in (A) do not match colours in (B) and (C) panels. ^a^Nursery trees derived from seeds collected in seed zone 404 were not available at nurseries at the time of sampling. ^b^The Sence Valley planted population derives directly from the Orchard 1 and therefore shares multiple seed provenances with it. ^c^The Hamsterley population is in seed zone 302 but is included as a seed source representative for the neighbouring zone 204.

Sampling of oak and birch was guided by the seed zone system established by Herbert et al. (1999), which assigns numerated zones to indicate the provenance of tree populations used for seed collection in commercial production (seed sources). For each species, we sampled four recent naturally colonised sites on former arable land adjacent to mature forests (putative parental stands), as well as three planted populations. We selected planted populations derived from seed stocks of known seed zone provenance, allowing the inclusion of matching samples from both seed sources and nursery‐grown seedlings. To account for variation in growth rate between sites, colonisers at each naturally colonised site were categorised into two groups of equal size (seedlings and saplings) based on diameter at breast height (DBH) and/or height. This approach enabled comparative analyses of genetic diversity across three cohorts representing the two afforestation strategies considered here: (i) adult trees from parental stands and seed sources; (ii) seedlings from natural colonisation sites and nurseries; and (iii) saplings from natural colonisation sites and planted areas (Figure 1A,B). To identify appropriate sites, we reviewed 51 potential natural colonisation sites and 55 planted sites. Sites were excluded if they lacked sufficient abundance of the target species, had prior land uses other than arable, were located too close to planted populations (to avoid genetic admixture), contained populations planted too long ago or had uncertain origins. Planted sites were also excluded if no corresponding nursery‐grown seedlings were available (Table S2A,B). Information about former land use, planting times and seed zone origin of commercially produced trees was obtained from land managers, seed suppliers and nurseries and master certificates when available. The final dataset included 42 sampling groups and leaf tissue samples for whole‐genome sequencing from 459 pedunculate oaks and 625 silver birches (Figure 2A; Table 1; Tables S3 and S4). The names of the nursery companies and the orchard have been anonymised, but together they represent a very significant proportion of UK grown stock. Details on per species sampling locations are provided in the ‘Extended materials and methods’ (Appendix S1).

**TABLE 1:** Geographic sampling of wild (WL) and commercially produced (CP) groups of pedunculate oak and silver birch in the United Kingdom. The column ‘*N*’ represents the number of individuals analysed after filtering for missing data. For nurseries, seed provenance regions of sampled trees are shown in parentheses.

Species	Type	Site	Pipeline group	Cohort	*N*	Latitude	Longitude
Oak	WL	Home Farm	Parental	Adults	21	51.17	−1.07
			Colonisers	Seedlings	24		
				Saplings	24		
		Slindon	Parental	Adults	23	50.89	−0.64
			Colonisers	Seedlings	26		
				Saplings	26		
		Barnett's Wood	Parental	Adults	28	51.09	0.53
			Colonisers	Seedlings	25		
				Saplings	25		
		Rumstead Road	Parental	Adults	17	51.30	0.63
			Colonisers	Seedlings	22		
				Saplings	21		
		Richmond Park	Seed source	Adults	56	51.44	−0.27
	CP	Nursery 1 (405)	Nursery	Seedlings	18	52.93	−2.79
		Nursery 2 (405)	Nursery	Seedlings	27	55.79	−2.12
		Barnetts Wood	Outplanted	Saplings	25	51.09	0.53
		George Henry Wood	Outplanted	Saplings	28	52.74	−0.59
		Church Road	Outplanted	Saplings	23	51.29	0.65
Birch	WL	Slindon	Parental	Adults	29	50.89	−0.64
			Colonisers	Seedlings	26		
				Saplings	26		
		Hainault Forest	Parental	Adults	24	51.63	0.15
			Colonisers	Seedlings	25		
				Saplings	25		
		Nashenden	Parental	Adults	16	51.35	0.49
			Colonisers	Seedlings	22		
				Saplings	21		
		Arger Fen	Parental	Adults	19	51.99	0.83
			Colonisers	Seedlings	26		
				Saplings	26		
		Spinningdale	Seed source	Adults	30	57.89	−4.26
		Elgin	Seed source	Adults	30	57.42	−3.38
		Dolforwyn Woods	Seed source	Adults	22	52.55	−3.24
		Presteigne	Seed source	Adults	30	52.24	−3.05
		Hamsterley	Seed source	Adults	30	54.70	−1.86
	CP	Orchard 1	Orchard	Adults	37	52.92	−2.93
		Nursery 2 (201)	Nursery	Seedlings	29	55.79	−2.12
		Nursery 2 (204)	Nursery	Seedlings	27	55.79	−2.12
		Nursery 2 (304)	Nursery	Seedlings	25	55.79	−2.12
		Sence Valley	Outplanted	Saplings	29	52.70	−1.42
		George Henry Wood	Outplanted	Saplings	26	52.74	−0.59
		Barnett's Wood	Outplanted	Saplings	25	51.09	0.53

To address the complexity of this dataset, we clarify here the terminology used throughout the text. The term ‘group’ refers to any set of samples within cohorts and afforestation pipelines and is used contextually. The terms ‘(sampling) site’ and ‘population’ are used similarly, but to avoid confusions deriving from counterintuitive meanings, we used them to refer to all trees in a natural colonisation site (regardless of cohort), seed sources and planted populations, but not nurseries. Cohorts consist of ‘adults’, ‘seedlings’ and ‘saplings’. In the natural colonisation pipeline, parental trees are categorised as adults, while colonisers are classified as either seedlings or saplings, as explained above. In the planting pipeline, seed source and orchard trees are considered adults, nursery trees are classified as seedlings and outplanted trees as saplings. The term ‘wild’ is applied to all groups within the natural colonisation pipeline as well as seed sources, except for the orchard, which is considered an outplanted population. ‘Commercially produced’ refers collectively to nursery seedlings and outplanted saplings, whereas ‘(out)planted’ specifically denotes outplanted trees. In analyses comparing groups of wild and commercially produced trees, a categorical variable ‘Type’ (‘wild/commercial’) is used to classify them. Finally, while the term ‘seed source’ is used throughout the text, it is important to note that these represent proxies, as precise information about the specific stands where seeds were collected was unavailable.

### 2.3 DNA Extraction, Resequencing and Variant Calling

Genomic DNA was extracted following a modified CTAB method (Doyle 1990; Inglis et al. 2018). Briefly, leaf tissue was ground and suspended in a sorbitol buffer for osmotic lysis. The lysate was then treated with a CTAB buffer containing 0.2% β‐mercaptoethanol to remove polysaccharides and proteins. The DNA was purified by chloroform alcohol (24:1) extraction and subsequently precipitated with sodium acetate and isopropanol.

Paired‐end (PE) 150 bp libraries with an insert size of 300 bp were prepared and sequenced on a DNBseq platform. A total of 14.18 and 10.96 billion PE reads were produced for oak and birch respectively, for an initial mean coverage per sample of ~12×. Read quality was evaluated using FASTQC (Andrews 2010). Trimming and quality filtering were conducted using Trim Galore version 0.6.10.

Reads were mapped using the mem algorithm in the Burrows‐Wheeler Aligner (BWA; Li and Durbin 2009) version 0.7.18 against the pedunculate oak reference genome dhQueRobu3.1 (RefSeq: GCF_932294415.1) available at NCBI, and the silver birch reference genome with ID 35080 (Salojärvi et al. 2017) as available in CoGe (Lyons and Freeling 2008). Read groups were assigned and BAM files generated with Picard Tools version 1.126 (http://broadinstitute.github.io/picard). Duplicates were marked also with Picard Tools. We used the HaplotypeCaller + GenotypeGVCFs tools from the Genome Analysis Toolkit (GATK; McKenna et al. 2010) version 4.4.0 to produce a set of SNPs in the variant call format (vcf). Samples presenting more than 20% of missing data were discarded at this point. Using vcftools version 0.1.16 (Danecek et al. 2011), we retained biallelic SNPs excluding those out of a range of coverage between 8 and 50, presenting a minor allele frequency (MAF) below 0.01 or with a genotyping phred quality score below 30. We then applied GATK generic hard‐filtering recommendations (GATK Best Practices; Van der Auwera et al. 2013; DePristo et al. 2011). Trimming and hard‐filtering parameters are provided in the ‘Extended materials and methods’ (Appendix S1).

To attain higher confidence in heterozygous SNP calling, we also implemented a minimum number of reads supporting each of the reported alleles or allelic depth (AD) > 3. One oak and four birch individuals were then identified as potential triploids based on allelic depth ratio, likely due to spontaneous polyploidisation or errors in species identification and were thereby removed from these initial SNP matrices. For most analyses (see below) we also removed positions with < 75% of individuals genotyped for each population with vcftools. The resulting datasets, named ‘Full datasets’ hereafter, consisted of 441 individuals and 1,491,996 SNPs for oak; and 611 individuals and 619,369 SNPs for birch. Further filtering and customisation were conducted for each specific analysis (Table S5).

### 2.4 Population Structure Analyses

To explore genome‐wide patterns of population structure, we used the program ADMIXTURE (Alexander et al. 2009). Prior to the analysis, SNP loci under linkage disequilibrium (LD) were filtered out from the ‘Full datasets’ with bcftools version 1.12 (Danecek and McCarthy 2017) using a *r* ^2^ limit of 0.4 in windows of 1 kb, resulting in a final data matrix of 777,742 SNPs in the case of oak and 323,245 SNPs in birch. We ran ADMIXTURE five times per *K* value, with *K* ranging from 2 to 10. Similarity scores among runs and graphics were computed with CLUMPAK version 1.1.2 (Kopelman et al. 2015). Using the same SNP datasets, we conducted principal components analyses (PCA) with the R package SNPRelate version 1.26.0 (Zheng 2012).

### 2.5 Comparative Estimates of Genetic Diversity Across Cohorts in Planting and Natural Colonisation Pipelines

Observed heterozygosity (*H*_O_) and nucleotide diversity (*π*) were computed using the population program from Stacks version 2.63 (Catchen et al. 2013). To test for significant differences across ‘Cohorts’ (adults, seedlings, saplings) and between tree ‘Types’ (wild, commercial), we applied beta‐regression models with random effects to account for differences among sampling groups, using the R package ‘glmmTMB’ v1.9.15 (Brooks et al. 2017). Observed heterozygosity and nucleotide diversity were used as dependent variables in separate models for each species. Model selection was performed using Akaike information criterion corrected for small sample sizes (AICc) using the R package ‘MuMIn’ v1.48.4 (Barton and Barton 2015). Z values and associated *p* values are also reported. In addition, the program ADZE (Szpiech et al. 2008) was used to estimate allelic richness (*A*_R_) for each of the populations.

To examine how genetic variation is partitioned among cohorts and among sites within cohorts; and to calculate how much of the total variance is due to differences between cohorts and sites, we applied hierarchical analyses of molecular variance (AMOVA) as described in Excoffier et al. (1992) using the ‘pegas’ v1.2 R package (Paradis 2010). We grouped individuals into two levels: age class cohorts in natural colonisation and planting pipelines; and sites or groups within cohorts in natural colonisation and planting pipelines. Cohort was used as the top‐level hierarchical grouping. While using sampling site as the top level might have been more biologically meaningful for the natural colonisation pipeline (with parental adults, seedling colonisers and sapling colonisers as subpopulations) this approach was not directly comparable to commercially produced tree populations, where cohorts of the same origin do not occur in a single location, preventing such classification. We ran separate analyses for our entire SNP dataset and for gene data only for comparative purposes.

We estimated the genomic inbreeding coefficient for each group within each species using runs of homozygosity (ROH) (*F*_ROH_; Ceballos et al. 2018). SNP datasets used for ROH were filtered as described in the ‘Full Datasets’ pipeline, but without any LD or MAF filtering as recommend by Meyermans et al. (2020) (further details are provided in the ‘Extended materials and methods’ and Table S5).

### 2.6 Coancestry Patterns in Planted and Naturally Colonised Populations

We explored within and pairwise levels of coancestry among groups of both species with the program ChromoPainter implemented in fineSTRUCTURE (Lawson et al. 2012). ChromoPainter relies on the ‘painting’ algorithm by N. Li and Stephens (2003), which has been proposed to be more effective at estimating fine‐scale population structure than other clustering analyses or fixation indices (Lawson et al. 2012). To detect subtler signs of differentiation, and because this program relies on linkage disequilibrium to achieve more refined assignment of nearest‐neighbour relationships, LD filters were not applied in this analysis. Prior to the analysis, SNP datasets were phased using SHAPEIT2 version 2.904 (O'Connell et al. 2014) after filtering out samples with more than 10% of missing data as recommended by the authors. This resulted in matrices of 1,485,178 SNPs and 377 samples for oak; and 323,361 SNPs and 569 samples for birch. We ran the analysis with 100 K burn‐in and 100 K sampling iterations, and a thinning interval of 1000 to produce a matrix of pairwise individual coancestry estimates, which were averaged over groups. In addition, an analysis of variance (ANOVA) was conducted to test for statistically significant differences in coancestry levels among the groups. Tukey's Honest Significant Difference (HSD) test was employed as a post hoc analysis to identify pairwise group differences. For this test, pairwise comparisons between individuals were filtered to include only comparisons between individuals from different cohort groups within each of the pipelines: parental trees, seedling and saplings colonisers in the natural colonisation pipeline; and seed sources, nursery trees and outplanted populations in the planting pipeline.

### 2.7 Differential Selection in Afforestation Pipelines

We used genotype–‘environment’ association analysis to test for differential selection between planted and young coloniser populations, and to identify potential candidate genes. We applied a redundancy analysis approach as implemented in the ‘vegan’ 2.6‐10 R package. RDA is a multivariate linear method that models the relationship between multiple response variables and explanatory variables, assuming linear associations between them (RDA; Borcard et al. 2011; Legendre and Legendre 2012; Van Den Wollenberg 1977). RDA has been widely used in genotype–environment association studies, with its effectiveness supported in multiple reviews. It often outperforms other approaches for detecting subtle selection signals, particularly when selective pressures are weak (Bernatchez et al. 2024; Capblancq and Forester 2021; Forester et al. 2016, 2018; Jones et al. 2013; Rellstab et al. 2015). Thereby, RDA was chosen as the most appropriate method for our study system, where selective pressures derived from planting practices occur over a single generation and are expected to be limited. A partial redundancy analysis (pRDA) was run with ‘Pipeline’ as the categorical explanatory variable (‘planted/coloniser’), and the alternative allele counts at each variant position as response variables. Allele counts were derived from 1,475,893 SNPs and 263 samples for oak; and 525,881 SNPs and 268 samples for birch (filters: MAF < 0.01, missing data ≥ 0.25). The first two PCs of a PCA on the SNP data were entered as covariates to control for population structure. Following the procedure described in Forester et al. (2018), we used pRDA loadings to identify candidate genes potentially involved in divergent selection by applying a four standard deviation cutoff (two‐tailed *p* value = 6.33 × 10^−5^). We then used the reference genomes' annotations to survey our set of candidate loci and identify those located within functionally annotated genes.

To further validate the patterns observed in the pRDA, we ran PIXY (Korunes and Samuk 2021) to estimate the average number of nucleotide differences between groups (*d*_XY_), nucleotide diversity (*π*) and *F*_ST_ in windows of 1000 bp. Since the calculation of *d*_XY_ requires both variant and invariant sites, we generated new genomic datasets from the original BAM files using bcftools mpileup with default settings. We filtered positions with a read depth outside the range of 8–50 or with per‐population missing rates exceeding 75%. This filtering resulted in final datasets comprising 775.3 million and 336.1 million sites for oak and birch, respectively.

### 2.8 Tree Health Assessment of Natural Colonisers Versus Planted Trees

To investigate whether different afforestation approaches led to variation in tree health, we assessed six sites containing stands of both planted and naturally colonised saplings of a similar age cohort between July 2023 and August 2024, for either one or both study species. Sites featured a mix of broadleaf species established on former arable land adjacent to ancient woodland. Planting and natural colonisation occurred across all sites between 2004 and 2018, with two sites hosting both study species, three sites hosting only pedunculate oak and one only silver birch (Figure S1; Table S6A–C). At each site, the health of circa 30 planted and 30 naturally colonised trees per species was evaluated. Trees were selected to represent the full extent of the areas of planting and natural colonisation, which varied in size. We recorded tree height, browsing, damage (combining stripping and fraying inflicted by mammals), reductions in living crown density and pest incidence including oak powdery mildew and birch leaf spot (Figure S2). Full protocols for tree health assessment are provided in the ‘Extended materials and methods’ (Appendix S1).

To assess differences in health indicators between planted and young coloniser populations of oak and birch, we used generalised linear mixed models (GLMMs) with a binomial family and logit link function for binary variables, and cumulative link mixed models (CLMMs) with a probit link function for ordinal response variables. Sampling sites were included as a random effect to account for potential site‐level variability. ‘Pipeline’ (planted/coloniser) and tree ‘Height’ (a proxy for tree age) were fitted as explanatory variables. Health indicators were categorised into three binary response variables, ‘Browsing’ (0 = no browsing; 1 = browsing), ‘Damage’ (0 = no damage; 1 = damage) and ‘Leaf spots’ presence (0 = no presence; 1 = presence; only for birch); and two ordinal response variables, ‘Crown density reduction’ and ‘Mildew incidence’ (the latter only for oak), scored from 0 to 4 and 0 to 3, respectively. After removing observations with missing values, the final dataset consisted of 355 records for oak and 222 for birch. Model selection for each health indicator was based on the Akaike information criterion corrected for small sample sizes (AICc). We used the dredge function from the ‘MuMIn’ package to compare models with various combinations of fixed effects. ‘Planted/Coloniser’ and ‘Height’ effects on each health indicator were assessed by examining the estimated coefficients of the final models. Random effect standard deviation estimates were used to evaluate the degree of variation between sites. GLMMs were conducted using the ‘lme4’ v1.1‐36 (Bates et al. 2015) R package and CLMMs using the ‘ordinal’ v2023.12‐4.1 (Christensen 2023) R package.

## 3 Results

### 3.1 Bioclimatic Coverage of Commercial Tree Seed Stands Compared to Wild Distributions

**FIGURE 3:**
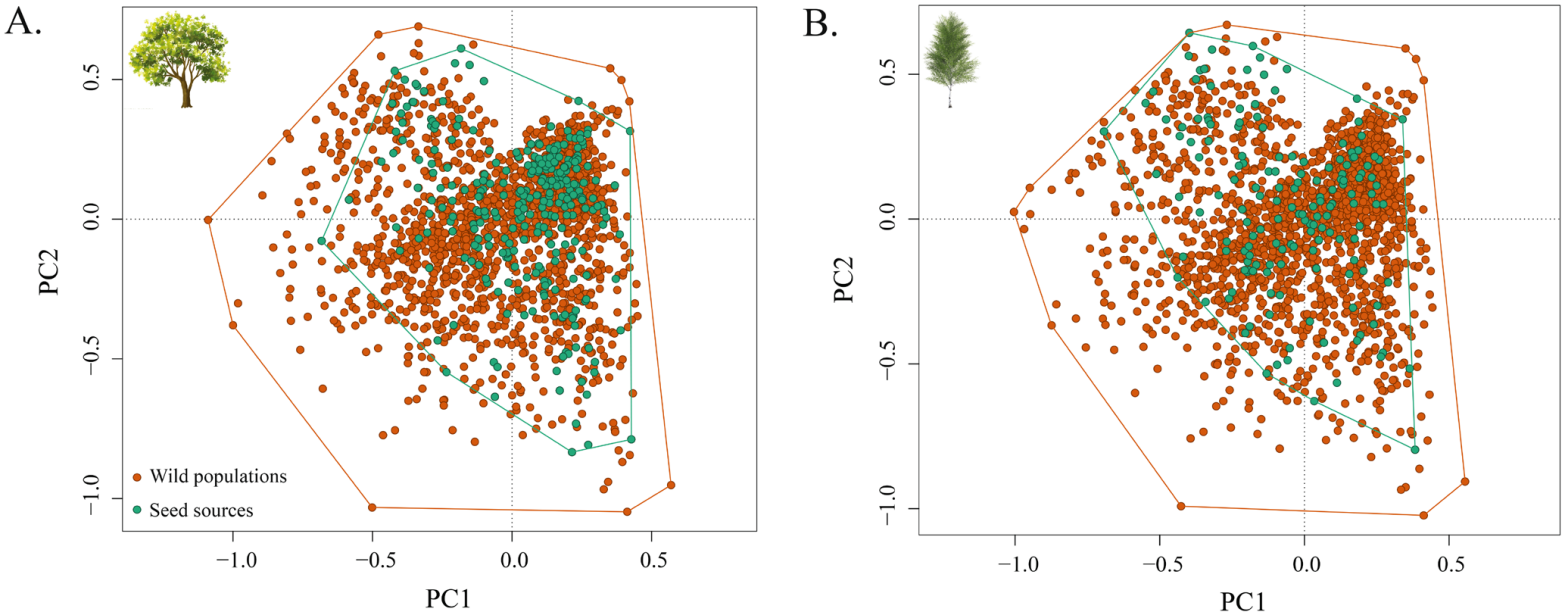
Bioclimatic envelope of commercial and wild tree distributions. Bioclimatic coverage differences for (A) *Quercus robur* and (B) *Betula pendula* , based on principal component analysis of georeferenced climate data.

The bioclimatic envelope, estimated as minimum convex polygons of PCA spaces derived from climatic variables, was smaller for seed stands compared to the species' wild distribution in all species included in the analysis. The represented polygon areas ranged from 2.5% ( *Tilia platyphyllos* ) to 75.2% ( *Quercus petraea* ) with an average of 31%. PERMANOVA indicated significant differences for 23 out of the 39 tested species, including our two study species, pedunculate oak and silver birch (Figure 3; Figure S1; Table S1; Appendix S2).

### 3.2 Population Structure

Population structure analyses based on genome‐wide, high‐quality independent SNPs revealed low levels of differentiation in both pedunculate oak and silver birch. ADMIXTURE analyses in oak revealed extensive shared ancestry among most sampled groups. Most individuals from the planted population of George Henry Wood and from Nursery 1 formed differentiated genetic groups. Several individuals at Slindon and Barnett's Wood, particularly colonisers, also showed signs of differentiation in distinct clusters (Figure 2B; Figure S4A). In birch, ADMIXTURE analyses identified parental trees and young colonisers from Slindon and Hainault Forest as differentiated groups. The planted population at Barnett's Wood showed shared ancestry with these groups. Most other wild populations, including seed source representatives, showed varying degrees of shared ancestry, except for Arger Fen parental trees, which formed a separate cluster. Additionally, substantial shared ancestry was observed among the Elgin seed source, two nurseries and the planted population at George Henry Wood (Figure 2C; Figure S4B). In the PCAs, variance explained by the first two PCs was low (0.84% and 0.78% for oak, 2.3% 0.73% for birch), and overlapping among groups was extensive (Figure S5A,B). An exception was the Arger Fen parental silver birch trees that clearly separated from the rest of the populations or groups, consistent with ADMIXTURE results. Follow‐up inquiries revealed that this site had been through a period of private ownership during which it may have been reforested with saplings of unknown, potentially non‐UK origin.

### 3.3 Genetic Diversity Across Cohorts in Planting and Natural Colonisation Pipelines

**FIGURE 4:**
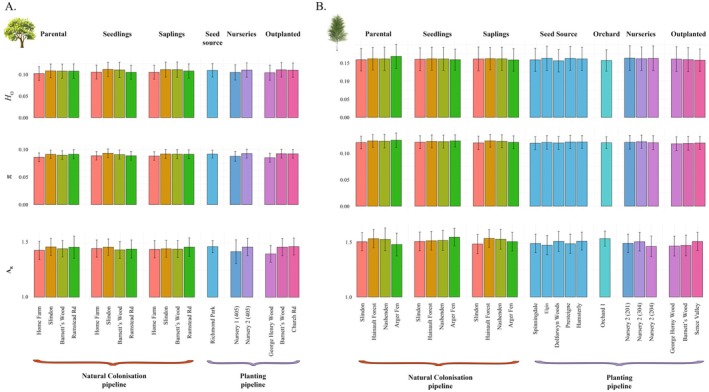
Genetic diversity indices. Estimates of observed heterozygosity (*H*_O_), nucleotide diversity (*π*) and allelic richness (*A*_R_) for cohort groups in natural colonisation and planting pipelines for (A) pedunculate oak and (B) silver birch. Whiskers represent variance.

Genetic diversity parameters within species were similar among age cohorts and between afforestation pipelines across all groups, with ranges of *H*_O_ = [0.102–0.112] and *π* = [0.085–0.093] for oak, and *H*_O_ = [0.158–0.161] and *π* = [0.120–0.124] for birch. Differences in *A*_R_ were also limited (*A*_R_ = [1.501–1.621] for oak; *A*_R_ = [1.468–1.554] for birch) (Figure 4; Table S3).

In our beta‐regression analyses testing for differences in genetic diversity, models including only ‘Cohort’ as the explanatory variable were consistently identified as the best fit based on AICc scores; although models including ‘Type’ also performed comparably (ΔAICc < 2). Naturally colonised seedlings and saplings exhibited significantly positive but small coefficient estimates compared to the reference group of adults, indicating modest increases in diversity metrics in younger cohorts. In oaks, *H*_O_ estimates were equal to 0.032 and 0.025 for seedlings and saplings, respectively, while *π* estimates were equal to 0.034 and 0.025. In birch, *H*_O_ estimates were equal to 0.048 and 0.037 for seedlings and saplings, respectively, while *π* estimates were equal to 0.058 and 0.044. In contrast, commercially produced trees consistently showed negative coefficient estimates relative to wild populations, but these effects were not statistically significant. Variation across sites, estimated via random effect standard deviations, was relatively low for both metrics (SD equal to 0.112 and 0.126 for *H*_O_ and *π* in oak, respectively; 0.062 and 0.079 for birch) (Table S7).

A series of AMOVAs were conducted to assess how variance is partitioned across cohorts of the pipelines. For naturally colonised oak, we found no significant differentiation between cohorts (Φ_CT_ = 0.019); however, a small but significant variance (2.19%) partitioned among sites within cohorts (Φ_SC_ = 0.022). Most of the variance occurred within sites (98.09%), with between‐site differentiation scores being negligible (Φ_ST_ = −0.003). In planted oak, small yet significant variance was fractioned between cohorts (0.87%) and between groups within cohorts (0.43%) based on all the SNPs. Φ‐statistics showed very limited differentiation (Φ_CT_ = 0.009, Φ_SC_ = 0.004), and most of the variance occurred within groups (98.7%) which were nevertheless scarcely differentiated (Φ_ST_ = 0.013) (Table 2). The same analyses using only SNPs within annotated genes yielded highly similar results (Table S8).

**TABLE 2:** Analysis of molecular variance (AMOVA) based on full SNP datasets comparing natural colonisation and planting pipelines. Percentage of total variance (% Total), *p* values and Φ‐statistics are reported for three hierarchical ‘Levels’: (1) between cohorts, (2) between sites or groups within cohorts and (3) within sites or groups. For natural colonisation populations, the second hierarchical level ‘sites’ corresponds to sampling sites. For planted populations, the second hierarchical level ‘groups’ corresponds to the different seed sources, nurseries and outplanted populations sampled and compared in this study.

Pipeline	Levels	Pedunculate oak				Silver birch			
		% Total	*p*	Φ‐Statistics		% Total	*p*	Φ‐Statistics	
Natural colonisation	Between cohorts	−0.28	0.894	Φ_CT_	0.019	1.84	0.001	Φ_CT_	0.018
	Between sites within cohorts	2.19	0.001	Φ_SC_	0.022	0.92	0.001	Φ_SC_	0.009
	Within sites	98.09		Φ_ST_	−0.003	97.24		Φ_ST_	0.028
Planting	Between cohorts	0.87	0.005	Φ_CT_	0.009	0.26	0.094	Φ_CT_	0.003
	Between groups within cohorts	0.43	0.001	Φ_SC_	0.004	6.35	0.001	Φ_SC_	0.064
	Within groups	98.70		Φ_ST_	0.013	93.39		Φ_ST_	0.066

In the AMOVA for naturally colonised birch sites, a significant 1.84% of the variance was structured between cohorts, and Φ_CT_ was low (0.018). An also significant but lower 0.92% of the total variance occurred among sites within cohorts, with Φ_SC_ = 0.009. Most of the variance occurred within sites (97.24%), with between‐site differentiation Φ_ST_ = 0.028. In planting pipelines, structure among cohorts was not significant either when based on the entire SNP dataset or gene SNPs, with very low Φ_CT_ values (0.003 and 0.002 respectively). Variance occurring among groups within cohorts was low yet higher in both cases (6.35% and 7.14% respectively), as well as Φ_SC_ if compared to the rest of the AMOVAs (0.064 and 0.072). However, most of the variance occurred once again within groups (93.39% and 92.61% respectively). Differentiation was also limited but higher if compared to the above results (Φ_ST_ = 0.066 for the entire SNP dataset, Φ_ST_ = 0.074 for gene SNPs) (Table 2). The analyses based on gene SNPs showed the same pattern, with partition values among cohorts and among sites within cohorts and Φ‐statistics being slightly higher (Table S8).

**FIGURE 5:**
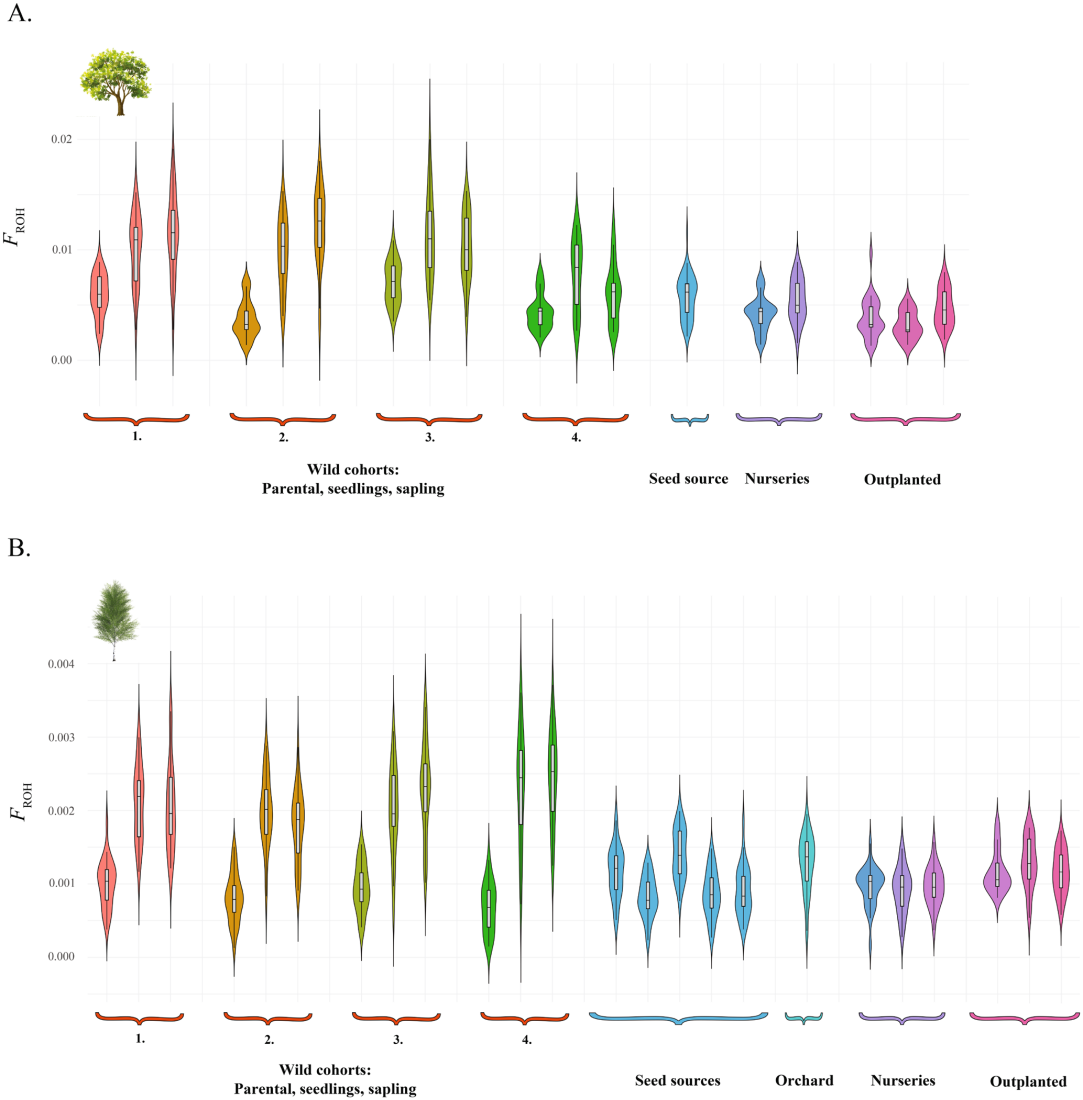
Inbreeding in wild and planted populations. Violin plots for inbreeding coefficient estimates based on runs of homozygosity (*F*_ROH_) across cohort groups in natural colonisation and planting pipelines. Estimates are shown for (A) oak and (B) birch populations. Natural colonisation sites are numbered for easy reference. Cohorts include parental, seedling and sapling groups for natural colonisation; and seed sources, nurseries and outplanted populations for planting pipelines. Violin width reflects the density of values.

Estimates of individual inbreeding coefficients based on runs of homozygosity were also computed. In oak, the analysis showed that average inbreeding in natural coloniser seedlings and saplings (*F*_ROH_ = 1.02 × 10^−2^) was double that of wild parental populations (*F*_ROH_ = 5.2 × 10^−3^) and planted cohorts (*F*_ROH_ = 4.5 × 10^−3^). A similar pattern was observed in birch, where the mean *F*_ROH_ for colonisers was 2.1 × 10^−3^, compared to 1.00 × 10^−3^ in wild parental and seed source populations, and 1.10 × 10^−3^ in planting cohorts (including orchard, nursery and planted populations) (Figure 5).

### 3.4 Coancestry Patterns in Planted and Naturally Colonised Populations

**FIGURE 6:**
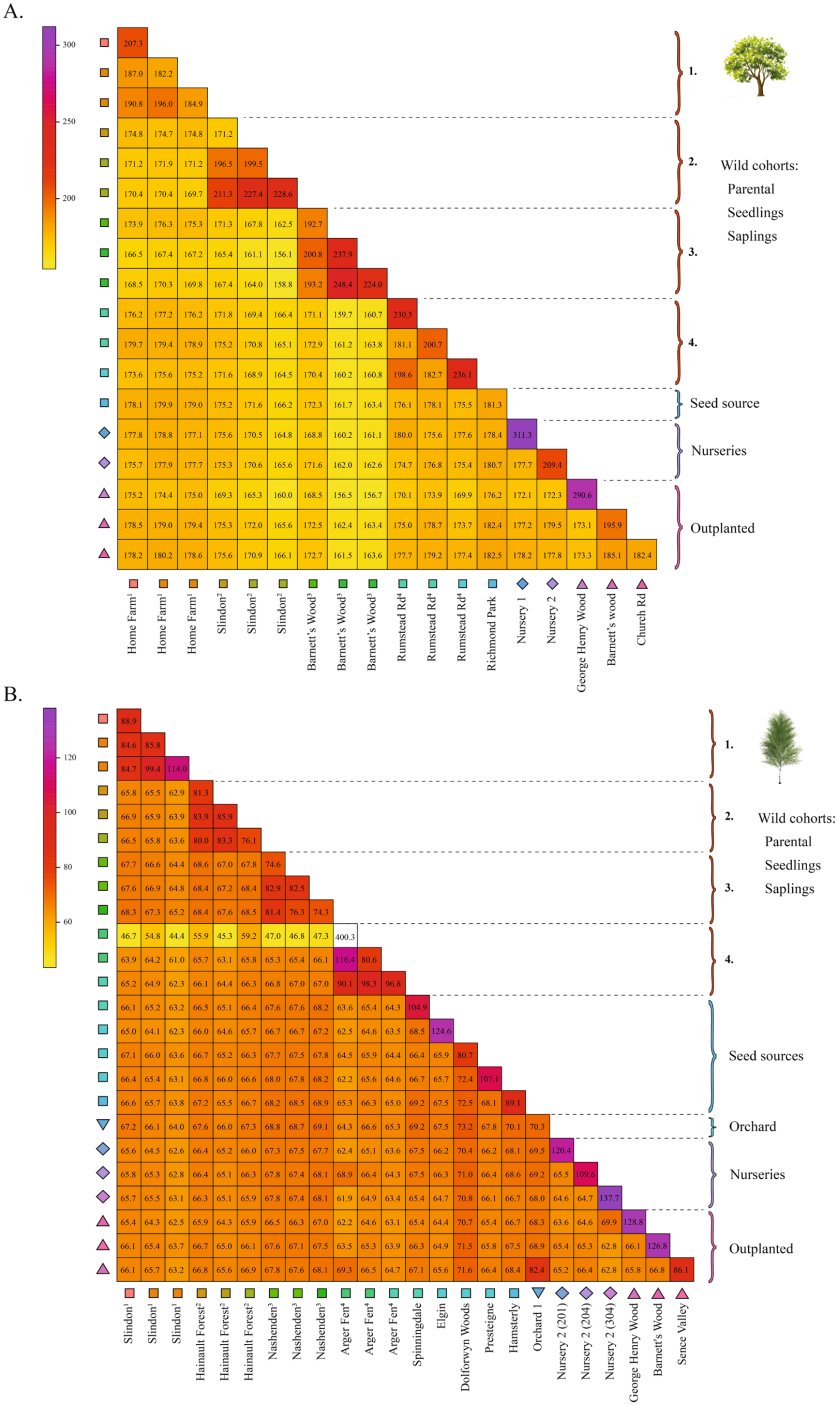
Coancestry patterns in planted and naturally colonised populations. Heatmaps of individual‐based within‐ and between‐group coancestry scores for (A) oak and (B) birch populations, computed using ChromoPainter. Natural colonisation sites are numbered for easy reference. In (B), the outlier Arger Fen parental cohort (shown in white) is excluded from the colour scale to improve visual clarity.

We further explored patterns of genetic diversity by calculating pairwise and within‐group coancestry scores using ChromoPainter, with the results visualised in heatmaps. First, as expected, we observed higher coancestry values between putative parental trees and neighbouring natural colonisers for both birch and oak. However, the highest within‐group coancestry values were found in nurseries and planted sites, particularly for birch, suggesting a common source within planting cohorts. Second, between‐group comparisons involving wild populations consistently showed lower coancestry scores compared to comparisons involving nursery and planted trees, suggesting lower differentiation between planted populations than between naturally colonised populations (Figure 6).

Congruently, an ANOVA followed by a Tukey's HSD post hoc test confirmed significantly higher between‐group coancestry scores in nurseries and planted sites compared to wild parental trees, seedling and sapling colonisers across all possible comparisons for oak (Table S9). This pattern may indicate some degree of genetic homogenisation among planted populations. The HSD test also indicated a decrease in coancestry scores among natural colonisation sites along the colonisation sequence. In birch, evidence for genetic homogenisation was less pronounced, although significantly or marginally significantly higher coancestry scores were detected for nurseries compared to parental trees, and for planted sites compared to parental, seedling and sapling colonisers. Additionally, coancestry between planted populations exceeded that between groups of nursery seedlings (Table S9).

### 3.5 Evidence of Selection in Afforestation Pipelines

**FIGURE 7:**
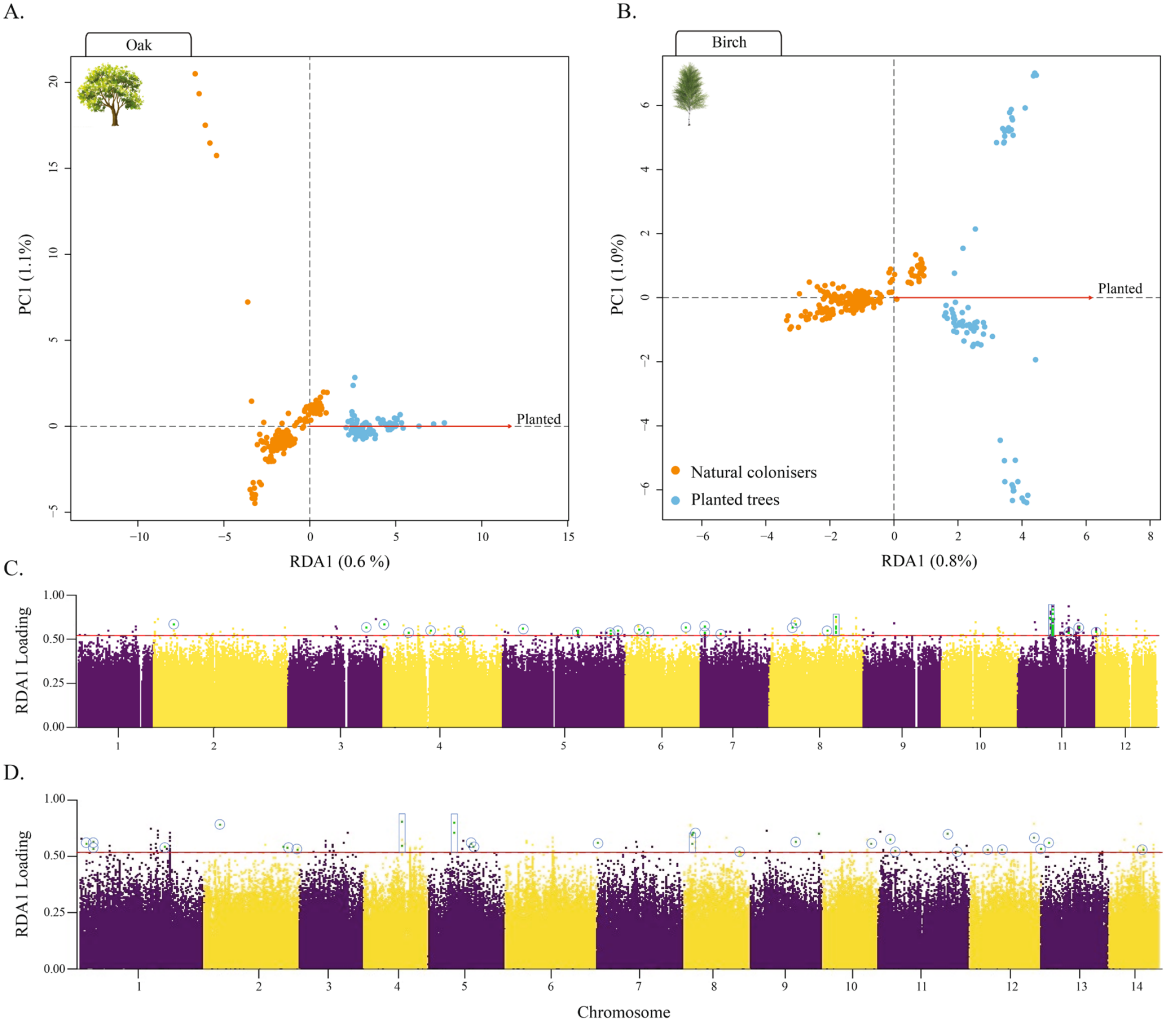
Allele enrichment in planted versus naturally colonised populations. Partial redundancy analyses controlling for population structure based on individual genotypes for natural colonisers (orange points) and planted populations (blue points) of (A) oak and (B) birch. Afforestation strategy (planted/not planted) was included as a single explanatory variable, shown as a red arrow indicating the direction and strength of association with the constrained RDA axis. The percentage of variance (unadjusted *R* ^2^) explained by each axis is shown in parentheses. Manhattan plots of per‐SNP RDA loading scores are also provided (C: for oak and D: for birch). SNP loadings were transformed as absolute deviations from the mean scaled by the maximum deviation, yielding values from 0 (near mean) to 1 (extreme). The dotted lines denote the implemented significance threshold of *p* = 6.33 × 10^−5^ used to identify candidate loci potentially under selection. Individual SNPs (circles) and neighbouring SNP sets (rectangles) of known function are shown. Function descriptions are provided in Table S11A,B and Figure S5.

An individual‐based partial RDA controlling for population structure revealed highly distinct association patterns for wild colonisers and planted saplings, resulting in two clearly separated clusters in both oak and birch (Figure 7A,B). Explained variance was significant in both species (*p* value < 0.001) yet limited: 0.28% in oak and 0.56% in birch (Table S10). In oak, 224 RDA SNP outliers were identified as candidate loci potentially under differential selection, with 60 of these SNPs located within 32 annotated genes with diverse functions (Figure 7C). Twenty‐six of the identified candidate SNPs were in a 600 kbp region of chromosome 11, encompassing several MADS‐box JOINTLESS‐like and SVP‐like genes. These genes are involved in abscission zone development and fruit and flower detachment (Mao et al. 2000), and in acting as repressors of flowering during the vegetative phase, playing a key role in flowering time regulation and response to environmental cues, especially temperature (Lee et al. 2007) (Figure S6A; Table S11A). In the case of birch, 141 candidate SNPs were identified, of which 26 occurred within genes of known function (Figure 7D; Figure S6B; Table S11B). PIXY did not identify any candidate loci at the top 5% windows of *d*_XY_, *F*_ST_ and *π* (Figure S7A,B), so that no overlap was observed with RDA candidate loci.

### 3.6 Differences in Phenotypic Tree Health Indicators Between Planted and Colonisers

In the GLMMs and CLMMs used to assess differences in health indicators between oak young colonisers and planted trees, the models identified by AICc scores as the best fit included only the explanatory variable ‘Type’ (Coloniser/Planted) for both ‘Browsing’ and ‘Damage’. However, for ‘Damage’, the model including ‘Height’ also performed similarly well (ΔAICc < 2). Random effect variation across sites was considerable for ‘Browsing’ (SD = 2.68). For ‘Mildew’ and ‘Crown density’, the best models included both ‘Type’ and ‘Height’, although for ‘Crown density’, the model excluding ‘Type’ was almost equally well supported (ΔAICc < 2). Coefficient estimates indicated significantly lower likelihoods of browsing (−1.65), damage (−1.78) and mildew (−0.27) occurrence in planted populations compared to colonisers. Random effect variation across sites for crown density was near zero; therefore, the model was refitted without random effects. The resulting coefficient for crown in planted populations was positive (indicating reduced health condition), but it did not reach statistical significance (Table S12).

For birch, the best models for ‘Browsing’, ‘Damage’ and ‘Leaf spot’ health indicators included both ‘Type’ and ‘Height’ as explanatory variables. No explanatory variables were consistently included in the best model for ‘Crown’ condition; however, the model including ‘Height’ showed similar performance based on AICc (ΔAICc < 2). Coefficient estimates suggested that planted birch populations have a significantly lower likelihood of browsing compared to natural colonisers (−0.88), but significantly higher likelihoods of damage (1.45) and leaf spot incidence (1.26). No significant effects were detected for crown density (Table S12).

## 4 Discussion

We found that genetic diversity in six planting afforestation pipelines was comparable to that observed across age cohorts of eight naturally occurring, recently established populations of pedunculate oak and silver birch in the United Kingdom. Encouragingly, this indicates that at the local level, tree planting can largely replicate natural populations as measured by genetic diversity indicators. Nevertheless, bioclimatic envelope comparisons indicate that the geographic location of registered seed stands may not fully capture the environmental variability found in the distribution of our study species and 21 other UK native species, potentially limiting adaptive diversity in planted populations. High levels of within‐group coancestry and moderate yet significant signs of between‐population genetic homogenisation were found in planted populations, particularly for oak. This suggests that at the landscape level, planted populations are more genetically similar to each other than naturally colonised populations, which may reduce differential local adaptation and risk future resilience. Genotype–environment association analyses also identified signals of divergent selection between natural colonisers and planted populations at a small number of ecologically relevant, functionally annotated loci. These findings, coupled with significant differences in health indicators, suggest that natural and actively afforested populations may be subject to differing selective pressures.

### 4.1 Similar Levels of Genetic Diversity and Low Differentiation Between Natural and Planted Populations

The lack of significant differences in genetic diversity when comparing naturally colonised versus planted populations suggests the absence of severe genetic bottlenecks in planting afforestation pipelines. It also refutes the hypothesised scenario of increased genetic diversity in nurseries and planted populations arising from multi‐origin composite seed batches or favourable early‐life conditions. Previous studies examining the impact on genetic diversity of different afforestation strategies have reported varied outcomes, from genetic depletion due to poor seed sourcing and bottlenecks related to nursery practices, to increased diversity in restored populations, often achieved through mixed‐origin plantings (for reviews see Jordan et al. 2019; Wei et al. 2023). In our study, naturally colonised sites and planted populations of both oak and birch were concentrated in the middle‐south and south‐east sections of Great Britain. However, while oak seeds for afforestation were locally sourced from matching or neighbouring seed zones (404 and 405), birch seeds originated from a more diverse range of regions, including direct collection from northern areas (regions 201 and 202) and a seed orchard pooling material from across the United Kingdom. Despite this broader seed provenance, conspicuous differences in genetic diversity between natural and planted populations or among planted populations themselves were not observed. This absence of diversity increases was particularly striking in mixed populations such as those from the Orchard 1 and the planted Sence Valley population. The low levels of geographic divergence among wild populations (Borrell et al. 2018; Petit et al. 2002) serving either as parental for new colonisers or seed sources, likely contributed to this lack of differentiation between sets of nursery seedlings and planted populations compared to natural ones.

These patterns have relevant implications for afforestation programmes in the United Kingdom. While local seed sourcing is often considered optimal to avoid maladaptation, time constraints and seed availability frequently necessitate more distant seed sourcing in afforestation programmes. In species like pedunculate oak and silver birch, characterised by wind‐pollination and long‐distance dispersal events resulting in generally low population differentiation, the negative impacts of using non‐local seeds might be negligible (Durka et al. 2017; Heenan et al. 2023; Höfner et al. 2022; Jia et al. 2020; Prakash et al. 2024). The low population differentiation in UK forests may facilitate commercial propagation, reducing complexity and challenges in seed sourcing (Whittet et al. 2016). However, population genetics analyses may overlook local adaptation processes involving specific loci, which could result in maladaptation if seed provenance is not carefully managed, as well as other risks like genetic homogenisation (see below).

### 4.2 Evidence of Recent Inbreeding in Naturally Colonised Populations

Estimates of the fraction of the genome covered by runs of homozygosity (*F*_ROH_) revealed evidence of potential inbreeding in colonised saplings and seedlings, a pattern observed in both oak and birch. In contrast, remaining wild populations (parentals and seed sources), as well as nursery and planted populations, exhibited generally lower inbreeding values. As a proxy of identity‐by‐descent (IBD) between haplotypes, *F*_ROH_ captures the total inbreeding coefficient of the individual, irrespective of factors such as generation overlapping or mixed origins in a given population (Ceballos et al. 2018). Patterns of increased inbreeding in recently established natural populations compared to planted ones have been previously reported, yet are uncommon in similar studies (Jordan et al. 2019; Wei et al. 2023). The elevated *F*_ROH_ scores in seedling and sapling colonisers compared to their corresponding parental populations suggest that the colonisation of former arable lands by neighbouring forest patches with potentially reduced effective population sizes may be resulting in inbred populations. It also suggests limited gene flow from more distant stands, potentially due to the extensive fragmentation and isolation of UK woodlands. Inbreeding due to small population sizes, as those resulting from habitat fragmentation, can cause substantial fitness reduction compared to outbred populations and elevate the risk of population failure (Neaves et al. 2015; Wright et al. 2008). These findings highlight the potential role of assisted gene flow and directed planting strategies to bolster genetic diversity and resilience in young woodlands of the United Kingdom. These strategies could mitigate the genetic risks posed by fragmentation and help sustain ecosystem services in the face of ongoing habitat loss and climate change (Cavers and Cottrell 2015).

### 4.3 Risk of Elevated Coancestry and Homogenisation in Planted Populations

In our study, we find contrasting patterns relative to potential homogenisation in oak and birch, likely driven by differences in seed sourcing. For oak, planted populations here analysed were in neighbouring seed provenance zones 405 and 402, with seeds sourced from also neighbouring zones 405 and 404. Natural colonisation sites used for comparison were located within seed provenance zone 405. AMOVA results revealed that the relative levels of genetic structure among sites within cohorts, although generally low, were over five times higher in naturally colonised sites than in commercially produced groups. This finding suggests that planting efforts may have contributed to genetic homogenisation relative to wild populations. Furthermore, HSD post hoc tests revealed significantly higher levels of coancestry between nurseries/outplanted populations compared to coancestry between wild cohorts. This pattern could arise from the extensive use of similar seed mixes across the landscape, as documented in other studies (Aavik et al. 2012; Breed et al. 2018; Höfner et al. 2022; Tong et al. 2020). However, coancestry scores also indicated high within‐group coancestry in nurseries, with Nursery 1 exhibiting the highest score in the dataset. Similarly, planted populations showed elevated within‐group coancestry levels, with the population in George Henry Wood ranking second overall. Together, these patterns suggest limited seed mixing during afforestation, with homogenisation in oak likely stemming from the repeated use of a small number of forest stands for seed sourcing.

For birch, the variation structured between groups within cohorts of commercially produced trees was nearly seven times higher than between sites within wild cohorts. Coherently, the ANOVA and HSD post hoc tests did not show a consistent pattern of heightened coancestry between nurseries and between outplanted populations as observed in oak, so that homogenisation is seemingly not occurring in actively afforested populations of silver birch in the UK, based on our dataset. However, similarly to oak, we observed high within‐group coancestry in nurseries and planted populations, including the George Henry Wood population, where seeds were reportedly sourced from geographically distant provenance regions 204 and 304. Once again, this pattern suggests that planting programmes tend to use material from a limited number of sources at a given site.

An important exception was observed in the Orchard 1 group, consisting of 32 individuals from six different provenance regions, which exhibited the lowest within‐group coancestry score in the dataset. The Sence Valley population, derived directly from this orchard, also showed low within‐group coancestry. The absence of homogenisation between planted populations, as observed in oak, despite similarly high coancestry in commercially produced trees, is likely due to a more diverse set of origins in used seeds and the lack of mixing for seedling propagation. This also explains the higher population structure between planted sites found in both the clustering analyses and the AMOVA for birch.

Despite the evidence of limited seed mixing and variety of stands used for seed sourcing in both oak and birch, our analysis of genetic diversity did not reveal a reduction in genetic standing variation in planted populations, as discussed above. However, the increased levels of within‐group coancestry observed in nursery and outplanted populations indicate a heightened risk of inbreeding in future generations once these trees begin reproducing. This raises concerns about potential genetic load and the long‐term viability of planted populations, as documented in similar studies (Jalonen et al. 2018; Prakash et al. 2024; Wei et al. 2023). Our results highlight that orchards can be valuable sources for mitigating increased coancestry in outplanted populations, thereby reducing the risk of inbreeding in subsequent generations.

### 4.4 Signals of Differential Selection Between Afforestation Strategies

Numerous studies have examined differences in genetic diversity between naturally colonised and planted woodlands (Jordan et al. 2019; Wei et al. 2023). However, tests for differential selection between afforestation strategies remain rare. Our RDA results revealed a clear separation between naturally colonised and planted individuals along a single significant axis for both species, yet with limited explained variance. This pattern indicates some degree of allele frequency differentiation between planted and naturally established populations, consistent with subtle signals of selection. Furthermore, several annotated loci were identified as candidate genes. In oak, a particularly relevant set of MADS‐box genes located on chromosome 11 is likely involved in flower and fruit maturation, as well as the timing of fruit detachment, traits that could be inadvertently targeted during seed collection. We note that human‐mediated selection of fruit detachment is remarkably similar to shattering‐related domestication traits frequently selected for in crops (Fuller and Allaby 2009). Furthermore, Conrady et al. (2023) reported evidence of a domestication syndrome involving flowering time and delayed shattering in plants cultivated for ecosystem restoration, a pattern that may parallel the genomic signal observed in our study species. Additional loci identified in our analyses are associated with responses to biotic and abiotic stressors, pathogen resistance, biosynthesis of waxes and other lipids, and various metabolic and regulatory functions (Figure S5; Table S11A,B). These candidate loci require further validation. Indeed, genome scans conducted using PIXY, which integrates intra‐group genetic diversity (*π*), inter‐group genetic differentiation (*d*_XY_), and *F*_ST_, did not recover the loci identified by RDA as potential candidates, underscoring the need for caution when interpreting these results. Future studies incorporating higher‐depth sequencing and common garden experiments will be essential to assess the biological relevance and adaptive impact of these signals. In particular, such approaches could help distinguish between directional selection acting on specific traits and potential selection release resulting from relaxed selective pressures under nursery or planting conditions.

### 4.5 Differences in Health Indicators Suggest Effects of Afforestation Strategies

A major question is whether observed patterns of homogenisation, inbreeding or selection have meaningful impacts on plant fitness and therefore population resilience. Further evidence of differential selective pressures between natural stands and planted populations was observed through analyses of health indicators. Planted populations of pedunculate oak exhibited a lower likelihood of browsing and other animal‐inflicted damage, such as stripping and fraying. They also showed reduced powdery mildew incidence and a reduction, though marginally significant, trend in crown health condition. Similarly, planted birch trees were less likely to suffer browsing; however, they appeared more susceptible to other forms of damage and leaf spot compared to their naturally colonised references.

Some of these effects, particularly the reduced incidence of browsing and physical damage, may be attributed to protective measures implemented during active afforestation efforts, such as individual tree guards or fencing of planted areas. The observed reduction in powdery mildew in oak populations may reflect differences in nursery conditions, where seedlings are often grown under controlled, pathogen‐reduced environments. Additionally, seed collection practices may favour healthier‐looking parent trees, inadvertently selecting for more resilient gene pools in planted populations. This interpretation aligns with our findings for oaks but contrasts with birch, where leaf spot incidence was higher in planted populations than in natural stands. Herbivory, pest incidence, and other health‐related pressures can vary considerably over time as newly established stands mature into woodland ecosystems. Previous studies have shown that mitigating these pressures in the early stages of afforestation may accelerate the transition of planted sites into more natural‐like forest conditions (Fuentes‐Montemayor et al. 2022). To fully characterise the long‐term differences in health status between natural colonisation and planted populations, further longitudinal analyses spanning multiple years will be necessary to account for time‐dependent variability in these indicators.

### 4.6 Implications for Resilient Afforestation Strategies

Future afforestation efforts are likely to be met with a combination of planting and natural colonisation. In light of our data, it is clear that tree‐planting pipelines can play a valuable role in afforestation, and in some cases mitigate genetic limitations in natural colonisation. Nevertheless, our analyses also identify opportunities to improve nursery pipelines. First, our analysis of wild adult populations underscores the importance of evaluating the quality of local forest patches as parental trees for natural colonisation or as seed sources, particularly considering past disturbances or management practices. We detected signs of inbreeding in naturally colonised populations, which likely derived from isolated stands occurring in fragmented landscapes. These populations may suffer from inbreeding depression, reduced fitness, or other consequences of small population sizes such as loss of diversity due to drift, making them suboptimal as parental populations for afforestation (Jalonen et al. 2018; Thomas et al. 2014). In this context, using saplings derived from diverse seed stocks obtained from genetically surveyed woodlands may enhance the viability and resilience of oak and birch forests in the United Kingdom, outperforming the benefits of natural colonisation strategies.

Second, and more broadly, our analyses based on remote sensing environmental data revealed potential mismatches between the ecological diversity of registered seed stands and the ecological breadth of natural distributions. Importantly, this analysis does not include unregistered sources and seed orchards, which are nonetheless widely used by seed collectors. Yet, such mismatches may lead to a loss of adaptive variability, potentially reducing the resilience of resulting woodlands, particularly under climate change scenarios (Havens et al. 2015). Addressing these mismatches will require coordinated efforts to ensure an ecologically representative seed supply through broader networks of seed collectors and established seed orchards. Improved infrastructure for seed storage and research aimed at overcoming seed storage and germination constraints may help mitigate interannual variation in seed availability (Broadhurst et al. 2016; Cavers and Cottrell 2015; Whittet et al. 2016).

Third, signals of increased consanguinity in commercially produced trees, as a result of few seed providers and nurseries supplying numerous afforestation sites, risk contributing to landscape‐scale genetic homogenisation over future generations, as well as concerns about inbreeding, resilience and genetic load in subsequent generations. To address this, seed suppliers and nurseries could expand seed sourcing protocols to include a wider range of origins, reducing genetic load risks and thereby producing more genetically diverse material to improve resilience to climate change and emerging pests and diseases. Although pooling seeds from multiple populations can also entail risks of maladaptation when seed sources span large geographic regions (Höfner et al. 2022; Tong et al. 2020), the apparent absence of strong neutral or adaptive genetic differences among UK populations of oak and birch allows for more flexible seed sourcing within reasonable limits. Our data suggest that the use of orchards may also be an effective strategy to prevent high levels of coancestry in actively afforested populations.

Fourth, although this study identified candidate loci potentially under selection, no evidence of strong, widespread selection or selective sweeps was detected at the genomic level between natural colonisation and planted tree populations. It is reassuring that current nursery practices appear effective at maintaining genetic diversity comparable to wild populations, yet our results highlight the importance of continued vigilance to avoid unintentionally introducing selection pressures during the production of planting material for conservation‐focused afforestation. Together, these findings emphasise the need for improved policies and support to meet the higher costs associated with producing genetically diverse planting material across the entire tree production pipeline, from seed collection to sourcing stock (potentially from multiple suppliers) at afforestation sites. Based on discussions with numerous stakeholders, we stress that enhancing tree production pipelines depends not only on nurseries themselves but also on supportive policies and informed consumer choices.

## Conflicts of Interest

The authors declare no conflicts of interest.

## Supporting information


**Appendix S1:** eva70146‐sup‐0001‐AppendixS1.docx.

**Appendix S2:** eva70146‐sup‐0002‐AppendixS2.pdf.

**Tables S1–S12:** eva70146‐sup‐0003‐TablesS1‐S12.xlsx.

## Data Availability

Resequencing data are deposited in the SRA database, Bioproject accessions: PRJNA1094567, PRJNA816149, PRJNA947136 and PRJNA825844. Per sample SRR accessions are provided in the Supporting Information. FRM and master certificates' data are available on request from the Forestry Commission. Climate data by the Met Office is available at https://catalogue.ceda.ac.uk/uuid/4dc8450d889a491ebb20e724debe2dfb/. Data on health indicators are provided in the Supporting Information.
