# Supplementary material for: Genetic Consequences of Tree Planting Versus Natural Colonisation: Implications for Afforestation Programmes in the United Kingdom
Source: Evol Appl. 2025 Aug 27;18(8):e70146. doi: 10.1111/eva.70146 (PMC12390626; doi:10.1111/eva.70146)
Supplement: Supplementary file 1 — Appendix S1: eva70146‐sup‐0001‐AppendixS1.docx. [file EVA-18-e70146-s002.docx]

**Genetic consequences of tree planting versus natural colonisation: Implications for afforestation programs in the UK**

Guillermo Friis, Nicola Cotterill, Nadia Barsoum, Marcia Webberley, Mohammad Vatanparast, Michael Charters, Rômulo Carleial, Richard Buggs, James S. Borrell

**SUPPORTING INFORMATION**

**Extended materials and methods**

*Study system and sampling design for comparative genomic analyses*

Pedunculate Oak - Oak sampling included four wild populations in seed zone 405, where putative parental trees and natural colonisers (seedlings and saplings) were sampled. Two nurseries providing seedlings from region 405 and three outplanted populations with saplings originating from seed zones 404 and 405, all located within seed zone 405, were also sampled. For seed source representatives, we included adult trees from a wild population in region 405. Since oak planting predominantly relied on seeds sourced from zone 405, parental stands from natural colonisation sites were also used as seed source representatives in subsequent analyses (Fig. 2A; Table 1).

Silver Birch - Birch sampling included four wild populations of putative parental trees and natural colonisers in seed zone 405, one nursery with seedlings sourced from three seed zones (201, 204, and 304), and two outplanted populations located in seed zones 402, and 405. These populations originated from seeds collected in zones 201, 204, and 304. Additionally, we sampled a seed orchard comprising 37 genotypes of plus trees originating from seed zones 107, 109, 204, 301, 302, and 401, as well as a planted population derived from this orchard, located in seed zone 403. Seed source representatives included five wild birch populations from regions 201, 302, and 304, corresponding to or closely aligned with the regions of origin of the outplanted saplings (Fig. 2A; Table 1).

*DNA extraction, resequencing and variant calling*Trimming and quality filtering were conducted using Trim Galore version 0.6.10 with parameters --stringency 20 --clip_R1 15 --clip_R2 15 -q 20 --length 90, resulting in a set of reads ranging between 90 and 135 bp long.

GATK generic hard-filtering parameters consisted of QualByDepth (QD) > 2.0; FisherStrand (FS) < 60.0; RMSMappingQuality (MQ) > 40; MappingQualityRankSumTest (MQRankSum) > -12.5; ReadPosRankSum (RPRK) < -8.0; and StrandOddsRatio (SOR) > 3.0 (GATK Best Practices; Auwera et al., 2013; DePristo et al., 2011).

*Population structure analyses*

ROHs for inbreeding coefficient estimates were computed using plink with parameters --homozyg-snp 50 --homozyg-kb 1000 --homozyg-density 50 --homozyg-gap 1000 --homozyg-het 2 in the case of oak, and --homozyg-snp 20 --homozyg-kb 100 --homozyg-density 50 --homozyg-gap 2000 --homozyg-het 2 in the case of birch.

*Tree health assessment of natural colonisers versus planted trees*Tree height was recorded using a Bosch measuring rod (2023), placed at the base of the tree and extended to the top of the leading stem. Heights were rounded to the nearest 0.5 m. Mammalian pressure was documented following Gill (1992), accounting for browsing and bark stripping/fraying up to 2 m around the base of each tree. Crown health was evaluated using a scoring system adapted from Innes (1990), Bussotti et al. (2002), and Redfern and Boswell (2004). This system assessed reductions in living crown density caused by shoot dieback, scored on a 0 – 4 scale, with 0 indicating no visible dieback (Fig. S2, Supp. Inf.).

Data on pest’s incidence was also collected. For pedunculate oak, ten shoots per tree were randomly selected from branches within 2 m of the ground, where access was unobstructed by bramble or other vegetation. If fewer than ten shoots were available, all accessible shoots were assessed. Oak powdery mildew (*Erysiphe alphitoides*) severity was scored on a 0–3 scale, with 0 indicating no visible infection. Severity levels corresponded to overall percentages of shoot infection (Fig. S2, Supp. Inf.). For silver birch, the presence or absence of leaf spots caused by *Anisogramma virgultorum* was recorded when more than 5% of leaves exhibited symptoms.

**Table S1.** Comparison of bioclimatic envelopes between commercial tree seed sources versus wild populations for 39 native UK tree species. The table reports the number of seed source data points from the UK Register of Basic Materials (UKRBM) and of surrogate points from Forest Reproductive Materials (FRM) master certificates, the total data points used, and the bioclimatic range areas (in PCA units) for wild and seed source populations. ‘Overlap’ represents the intersected area between wild and seed source ranges, ‘Represented %’ indicates the proportion of the wild range covered by seed sources and ‘Outside %’ refers to the proportion of the seed source range outside the wild range. PERMANOVA p-values indicate significant differences in bioclimatic envelope (*p < 0.05*).

**Table S2.** List of surveyed sites assessed for suitability in the experimental design for (A) pedunculate oak and (B) silver birch. Sites were reviewed for the presence of naturally colonised and planted populations, former land use, planting date, and species composition. The tables report site names, geographic coordinates, planting years (if applicable), species composition, and notes on exclusion criteria. Sites were excluded if they lacked sufficient abundance of the target species, had non-arable prior land use, were too close to planted populations (risking genetic admixture), had been planted too long ago, or had unknown origins.

**Table S3.** Sampled groups, number of genotyped individuals (N), and mean genetic diversity indices for wild (WL) and commercially produced (CP) populations of pedunculate oak and silver birch in the UK. Genetic diversity indices include observed heterozygosity (*H*_O_), nucleotide diversity (π), allelic richness (*A*_R_), and private allelic richness (*P*_R_), calculated using SNP markers. Variance is reported for all metrics. The column ‘N’ represents the number of individuals analysed after filtering for missing data. For nursery groups, the seed provenance regions of sampled trees are shown in parentheses.

**Table S4.** Sequenced samples with fieldwork and sequencing details. Identifiers, species, population names, afforestation pipeline (natural colonisation or planting), pipeline group, sequencing depth, missing data, geographic coordinates, tree size measurements [diameter at breast height (DBH) and height], assigned cohort, and NCBI SRR and Bioproject accessions are provided.

**Table S5.** List of genomic datasets used in the reported analyses and filters applied (in addition to GATK generic hard-filtering recommendations; Auwera et al., 2013; DePristo et al., 2011). Abbreviations LD and MAF and HWE denote linkage disequilibrium and minimum allele frequency, respectively. See the main text of the article for more information on applied filters.

**Table S6.**  Sampling sites (A) and health indicators data for tree health assessments in pedunculate oak (B) and silver birch (C). The tables include site names, GPS coordinates, the area (in acres) of planted and naturally colonised populations, and the estimated year of establishment for each population. Sites were selected based on their suitability for comparative health indicator analyses between planted and natural colonisation cohorts of similar age.

**Table S7.** Beta-regression models assessing the effects of ‘Cohort’ (adult, seedling, sapling) and ‘Type’ (wild versus commercially produced) on observed *H*_O_ and π in pedunculate oak and silver birch groups of trees. The ‘Models’ sections include the intercept, cohort, and type effects, along with degrees of freedom (d.f.), log-likelihood values, corrected Akaike Information Criterion (AICc), ΔAICc, and model weights. The ‘Coefficients’ sections provide estimated effects, standard errors (Std. Error), Z-values, p-values (Pr(>|z|)), and standard deviations of site-level random effects (Site (s.d.)) for top-ranking models. Cohort effects represent differences relative to adult trees, and type effects reflect differences relative to wild populations.

**Table S8.** Analysis of Molecular Variance (AMOVA) results based on full SNP datasets and SNPs within annotated genes, comparing natural colonisation and planting pipelines. Variance components (Sigma), percentage of total variance (% Total), p-values, and *Φ*-statistics are reported for three hierarchical ‘Levels’: (1) between cohorts, (2) between sites or groups within cohorts, and (3) within sites or groups. For natural colonisation populations, the second hierarchical level corresponds to sampling sites. For planted populations, the second level ‘groups’ includes seed sources, nurseries and planted populations.

**Table S9.** Tukey Honestly Significant Difference (HSD) tests on an ANOVA comparing mean pairwise coancestry values across wild and commercially produced trees for pedunculate oak and silver birch. Coancestry values were calculated from all pairwise comparisons of individuals and then filtered to include only comparisons between individuals from different populations within each group. Compared cohort groups were parental trees, seedling and saplings colonisers in the natural colonisation pipeline; and seed sources, nursery trees and outplanted populations in the planting pipeline. The table reports pairwise group comparisons, the estimated differences in mean coancestry values (Difference), the 95% confidence intervals for the differences (95 Low, 95 High), and adjusted p-values (P-adjusted) from the Tukey test. Statistically significant differences (P-adjusted < 0.05) are marked with an asterisk (*).

**Table S10.** Variance partitioning analysis of genetic variation in pedunculate oak (*Quercus robur*) and silver birch (*Betula pendula*) based on Redundancy Analysis (RDA). The table reports adjusted *R²* values (%) and p-values for the effects of afforestation type (Type: planted vs. coloniser), population structure (first principal components of a PCA based on genome-wide SNPs), and their combined effects. Conditional effects are also shown: ‘Type | Pop structure’ represents the variance explained by afforestation type after accounting for population structure, while ‘Pop structure | Type’ represents the variance explained by population structure after controlling for afforestation type. Significant p-values (*p* < 0.05) indicate that the explanatory variable significantly contributes to genetic variation.

**Table S11.** Candidate functional loci identified as outliers in partial Redundancy Analysis (pRDA) testing for divergent selection between planted and naturally colonised populations for (A) pedunculate oak and (B) silver birch. Lists of genes containing outlier SNPs, including gene names, chromosome identifiers, genomic coordinates (start and stop positions), functional descriptions, the number of SNPs detected per gene, and their specific positions are provided. Candidate loci were identified based on RDA loadings exceeding a four standard deviation threshold (p = 6.33 × 10⁻⁵) while controlling for population structure effects using the first two principal components of a PCA on SNP data.

**Table S12.** Generalised and cumulative link mixed model results for health indicators in pedunculate oak and silver birch. Health indicators include ‘Browsing’ and ‘Damage’ (binary variables), ‘Mildew’ (oak only; ordinal variable, 0–3 scale), ‘Leaf spots’ (birch only; binary variable), and ‘Crown density’ (ordinal variable, 0–4 scale). For both species, the tables report model comparisons based on corrected Akaike Information Criterion (AICc), including the model variables ‘Type’ (planted/coloniser) and ‘Height’ (proxy for tree age), degrees of freedom (d.f.), log-likelihood, ΔAICc (relative to the top-ranking model), and model weights. The top-ranking models (lowest AICc) are highlighted. The ‘Effects’ sections present estimated effects for the predictor variables ‘Type (Planted)’ and ‘Height’, along with their p-values (Pr(>|z|)) and the standard deviation of the random site-level effect (Site (SD)). Significant effects (p < 0.05) are indicated by asterisks.

**Figure S1.** Study site locations for planted versus naturally colonised *Quercus robur* and *Betula pendula* for health indicators analyses.


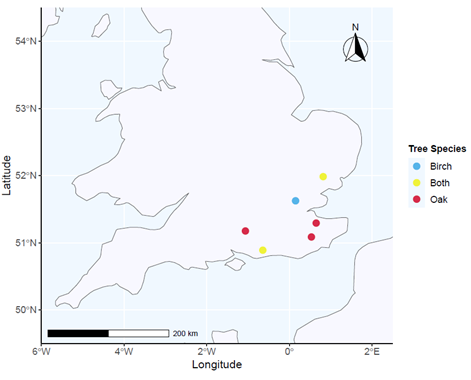


**Figure S2.** (A) Crown density scoring system assessed by the reduction in living crown density caused by shoot dieback. (B) Oak powdery mildew intensity score.

**
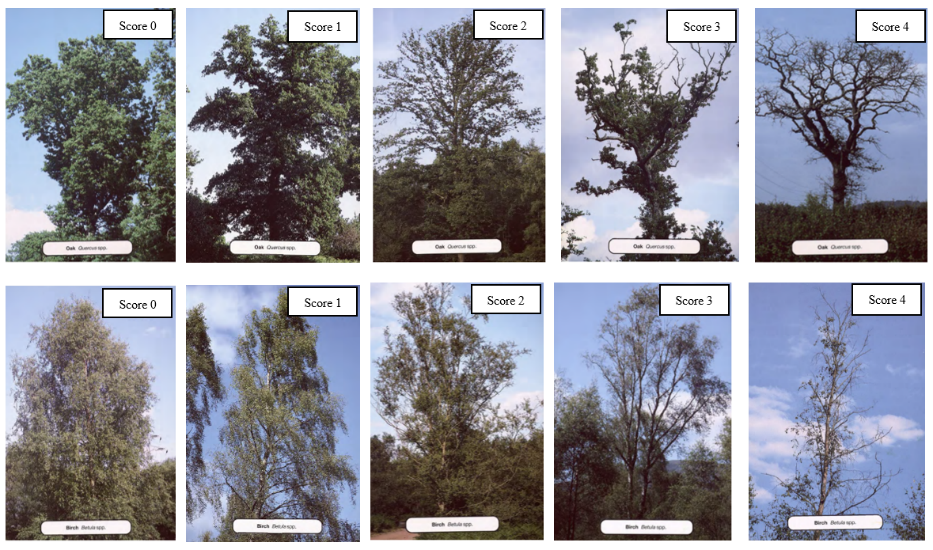
A.**

| Crown density (%) | Score |
| --- | --- |
| 0 - ≤ 5 | 0 |
| > 5 - ≤ 25 | 1 |
| > 25 - ≤ 50 | 2 |
| > 50 - ≤75 | 3 |
| > 75 - 100 | 4 |

**B.**


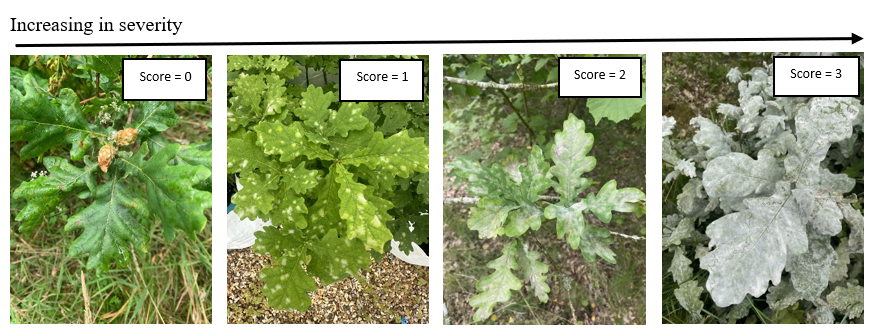


| Severity of infection on leaves (%) | Score |
| --- | --- |
| No infection | 0 |
| ≤25% | 1 |
| >25 ≤ 75% | 2 |
| >75 ≤100% | 3 |

**Figure S3.** Distribution of seed source forests for 15 native UK tree species. Each map shows the geographic locations of seed sources, with points representing registered sites obtained from the UK Register of Basic Materials (UKRBM). Grid reference data were converted to latitude and longitude coordinates using the UK Grid Reference Finder (UKGRF) and plotted for visual inspection. A total of 328 seed source sites were recorded across the 15 study species.


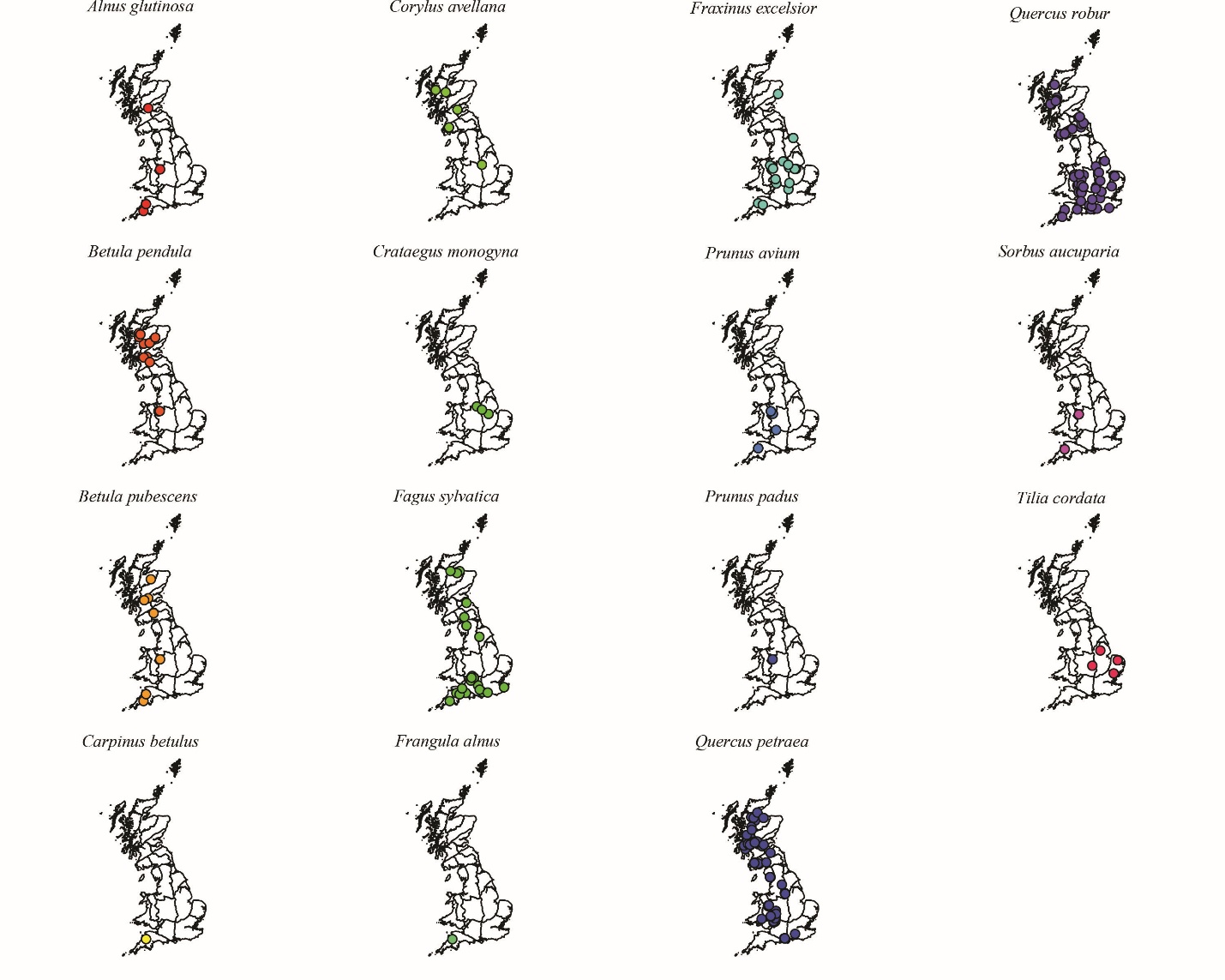


**Figure S4.** ADMIXTURE analysis for (A) oak and (B) birch populations. Ancestry proportions were estimated using ADMIXTURE for K values ranging from 2 to 6, based on 777,742 and 323,425 independent SNPs. Each plot represents individuals as vertical bars, with colours indicating the proportion of genetic ancestry assigned to each inferred cluster.


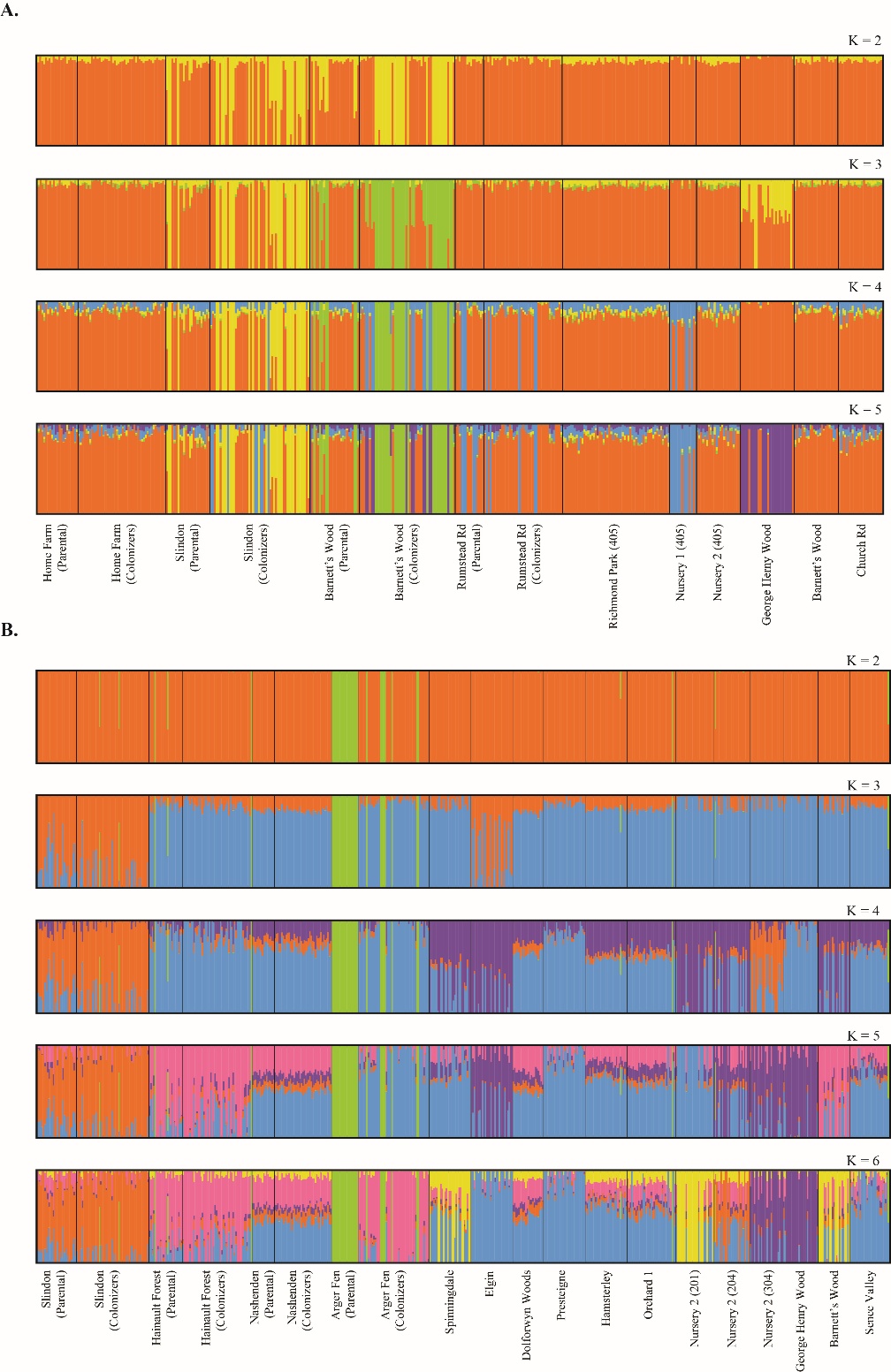


**Figure S5. P**rincipal component analysis (PCA) plots of PC1 and PC2 for oak (A) and birch (B), based on the same SNP datasets used for ADMIXTURE analyses. The percentage of variance explained by each axis is shown in parentheses.


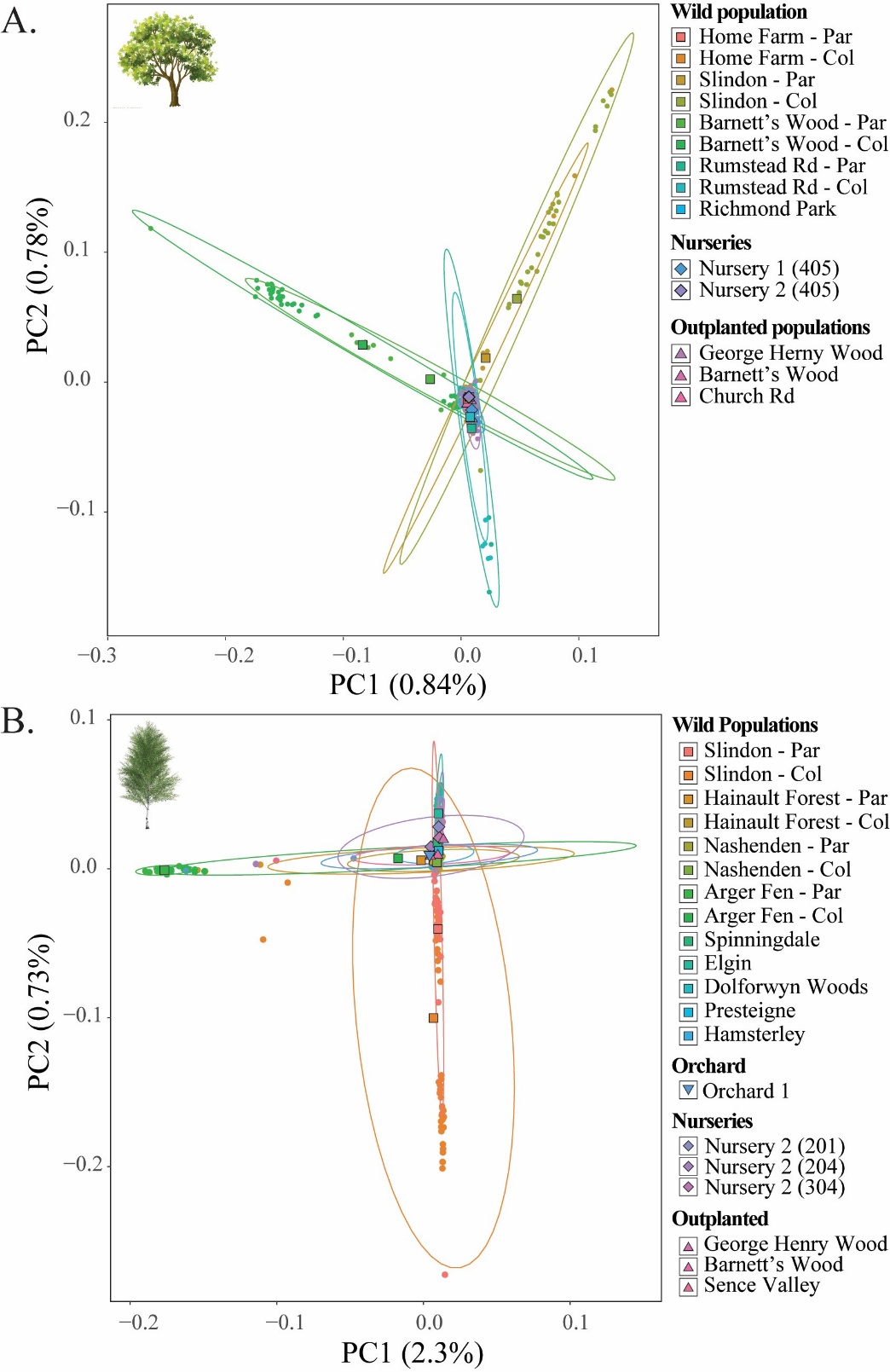


**Figure S6**. Signals of selection in the UK pedunculate oak (A) and silver birch (B) based on partial redundancy analyses and Manhattan plots of per SNP RDA loading scores. The dotted line denotes the implemented significance threshold of P = 6.33 × 10⁻⁵ to identify candidate loci potentially under selection. Known functions of candidate loci are reported in the figure.


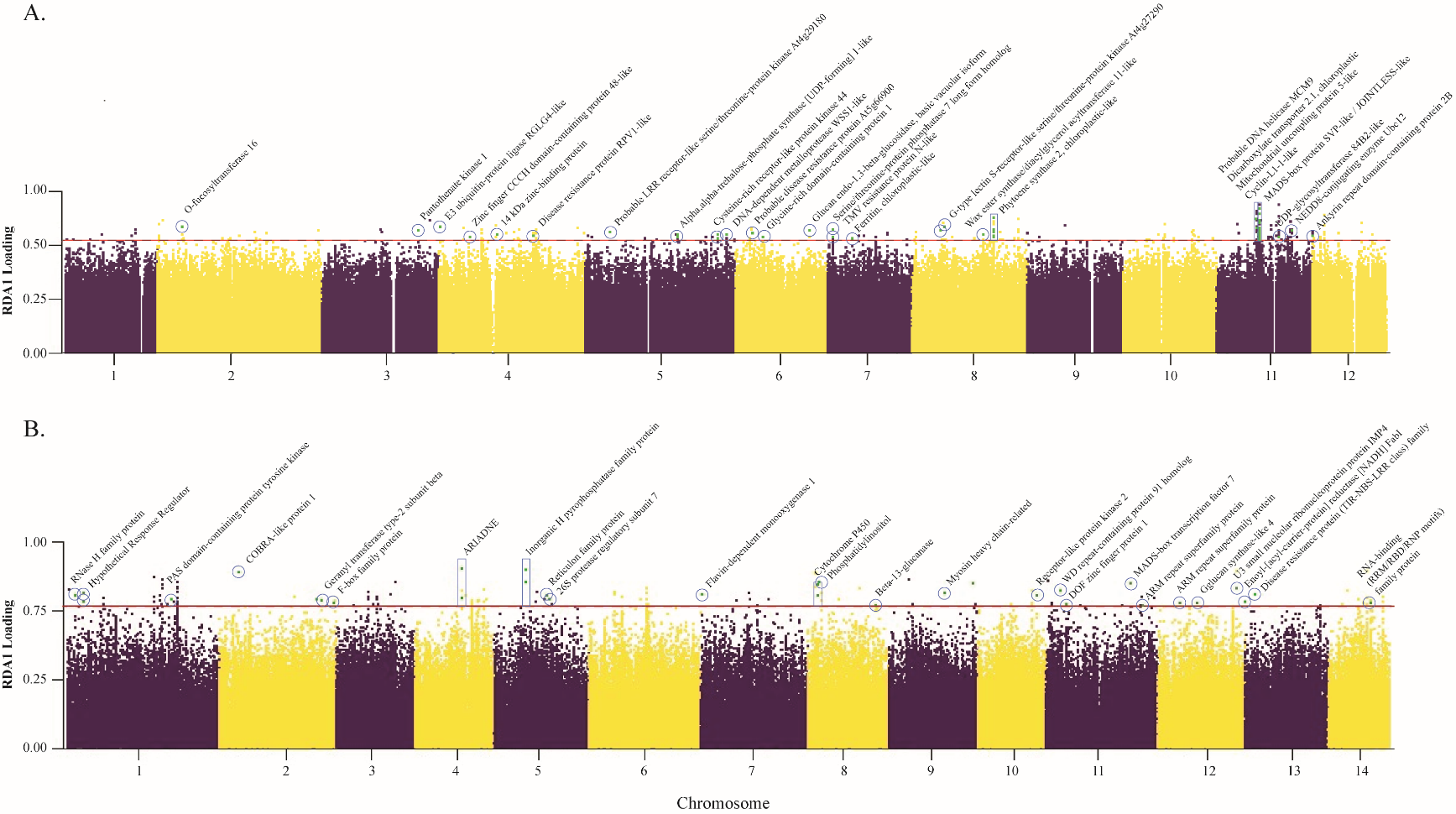


**Figure S7.** Genome-wide estimates of nucleotide diversity (*π*), absolute genetic divergence (*D*_XY_), and fixation index (*F*_ST_) for (A) oak and (B) birch populations based on PIXY analyses. Manhattan plots display per-SNP values across the genome Comparing planted and naturally colonised populations.

A.


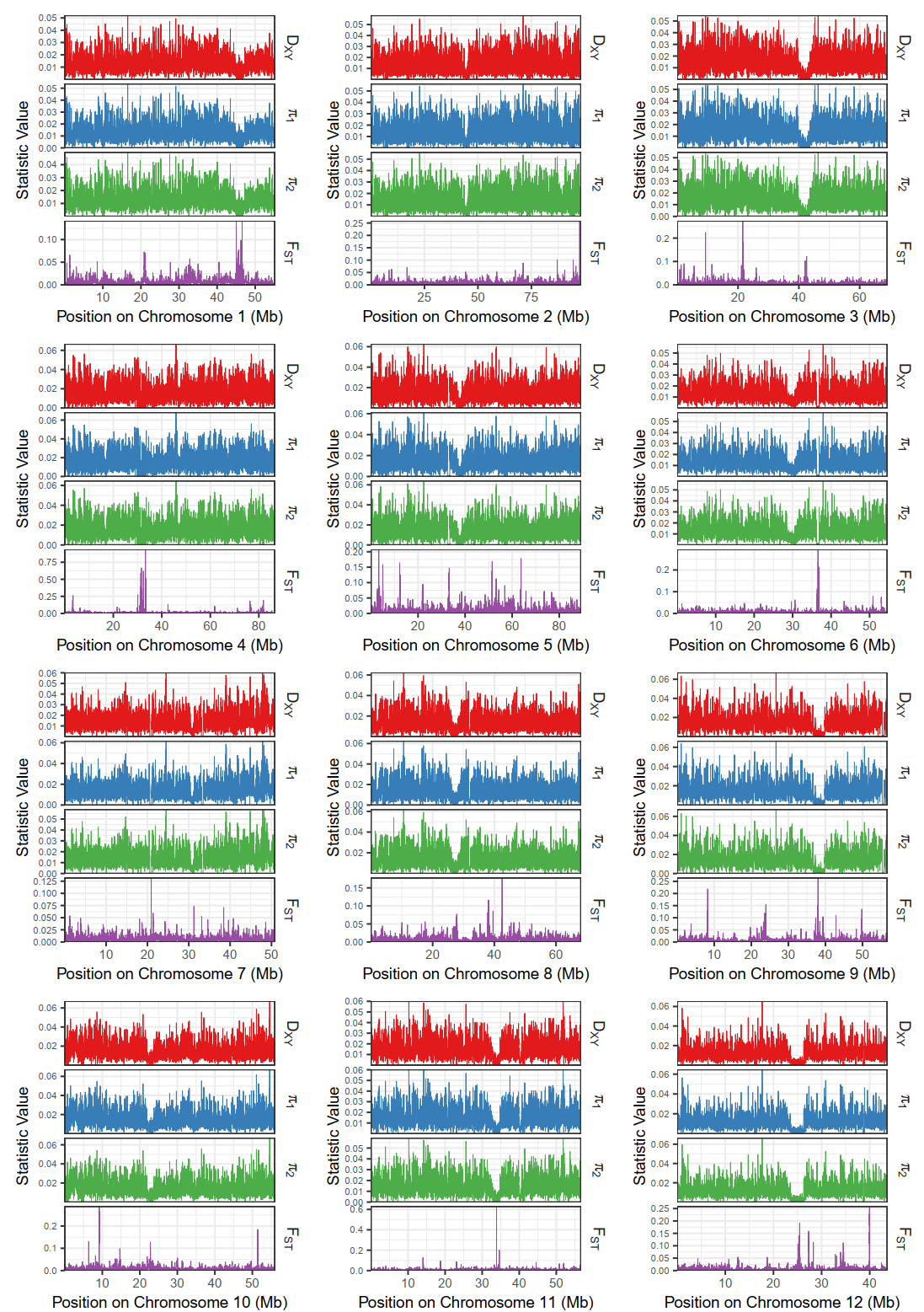


B.


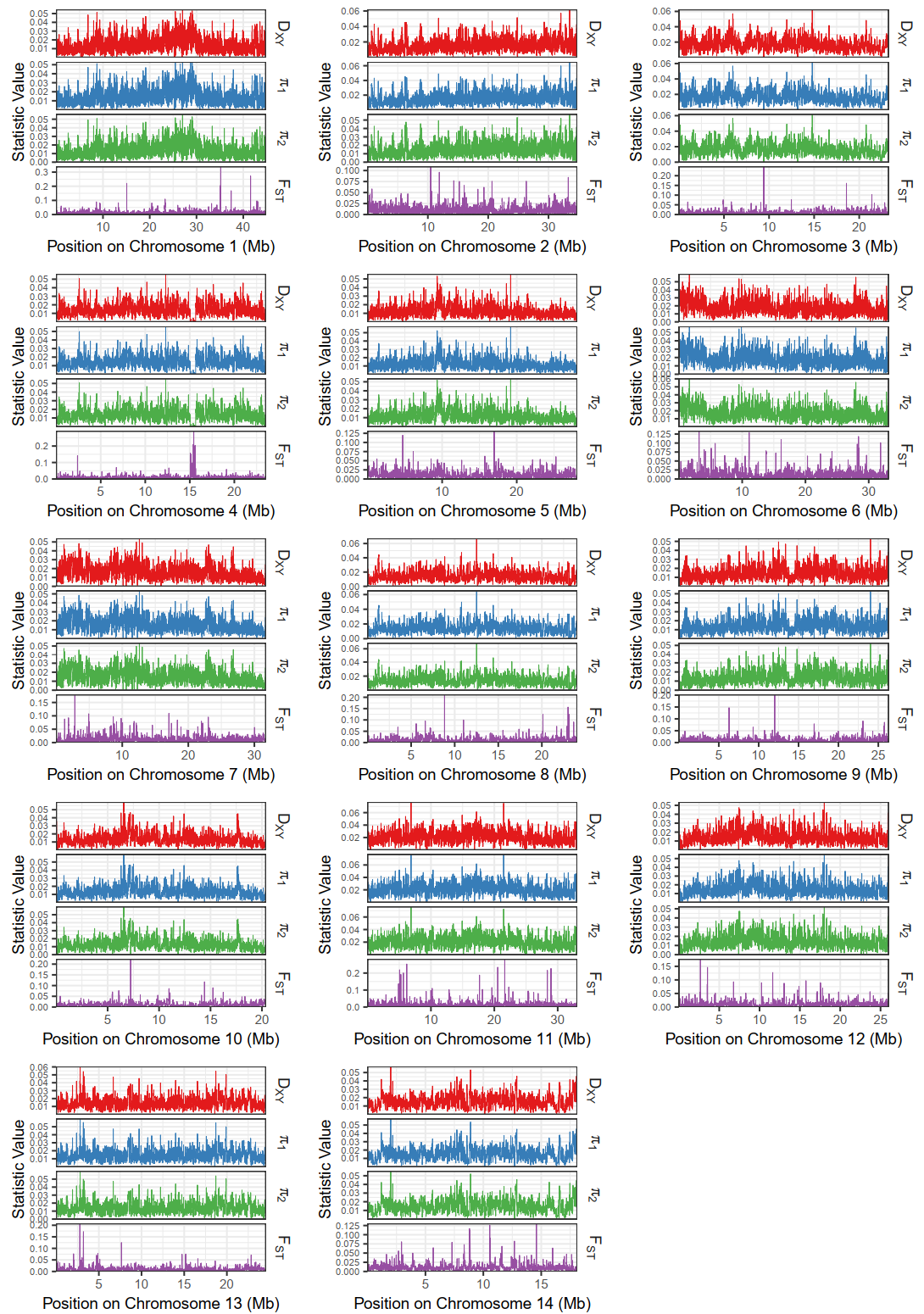


**References**

Auwera, G. A., Carneiro, M. O., Hartl, C., Poplin, R., del Angel, G., Levy‐Moonshine, A., . . . Thibault, J. (2013). From FastQ data to high‐confidence variant calls: the genome analysis toolkit best practices pipeline. *Current protocols in bioinformatics*, 11.10. 11-11.10. 33.

Bussotti, F., Cozzi, A., Ferretti, M., Cenni, E., Bettini, D., & Nibbi, R. (2002). Crown condition assessment at the CONECOFOR Permanent Monitoring Plots. *Journal of Limnology, 61*, 12-18.

DePristo, M. A., Banks, E., Poplin, R., Garimella, K. V., Maguire, J. R., Hartl, C., . . . Hanna, M. (2011). A framework for variation discovery and genotyping using next-generation DNA sequencing data. *Nature Genetics, 43*(5), 491-498.

Gill, R. (1992). A review of damage by mammals in north temperate forests: 3. Impact on trees and forests. *Forestry: An International Journal of Forest Research, 65*(4), 363-388.

Innes, J. L. (1990). Assessment of tree condition.

Redfern, D., & Boswell, R. (2004). Assessment of crown condition in forest trees: comparison of methods, sources of variation and observer bias. *Forest Ecology and Management, 188*(1-3), 149-160.
