# Supplementary material for: Genetic Consequences of Tree Planting Versus Natural Colonisation: Implications for Afforestation Programmes in the United Kingdom
Source: Evol Appl. 2025 Aug 27;18(8):e70146. doi: 10.1111/eva.70146 (PMC12390626; doi:10.1111/eva.70146)
Supplement: Supplementary file 2 — Appendix S2: eva70146‐sup‐0002‐AppendixS2.pdf. [file EVA-18-e70146-s003.pdf]

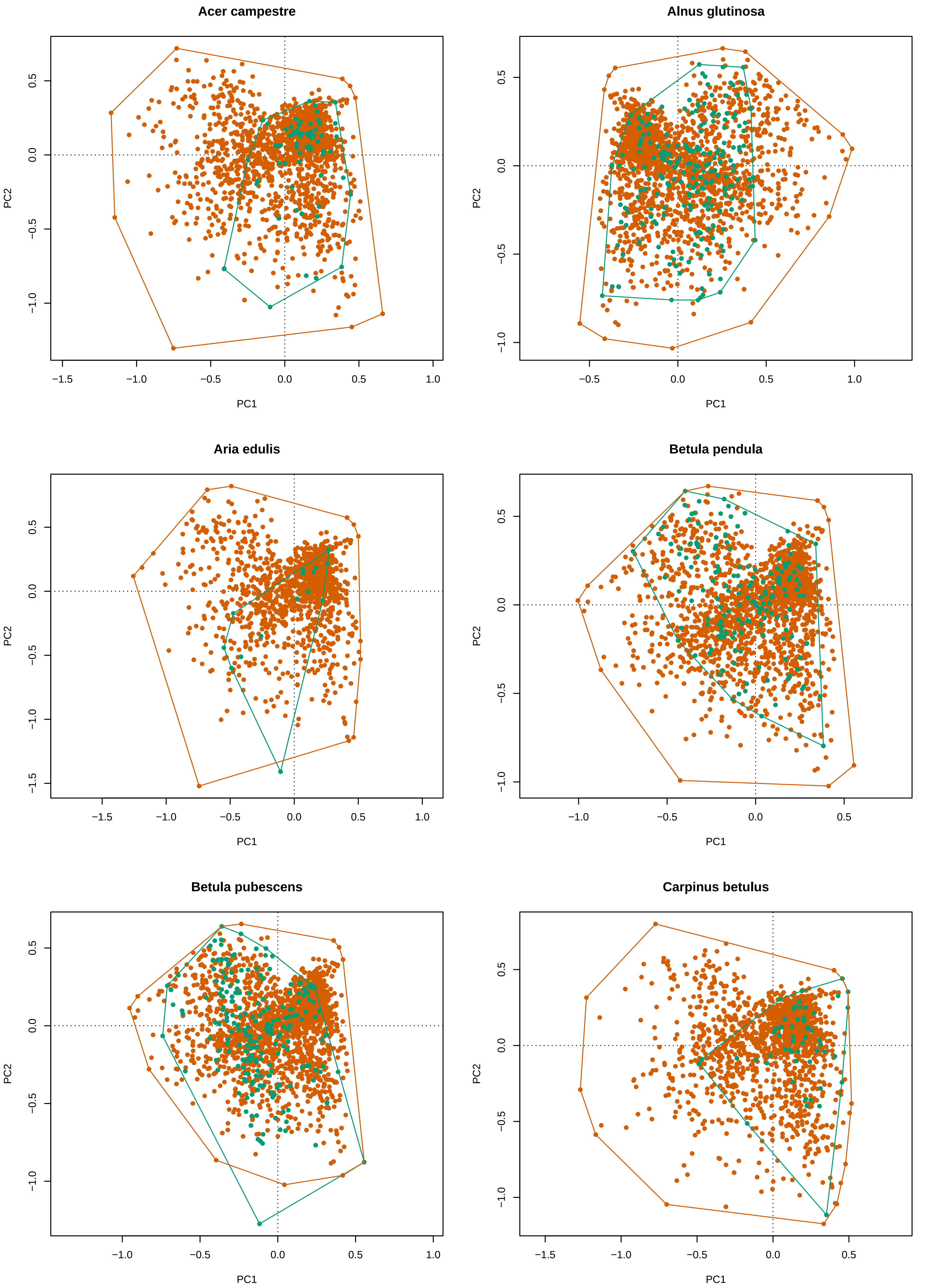

Acer campestre
Alnus glutinosa
Aria edulis
Betula pendula
Betula pubescens
Carpinus betulus
PC1
PC2

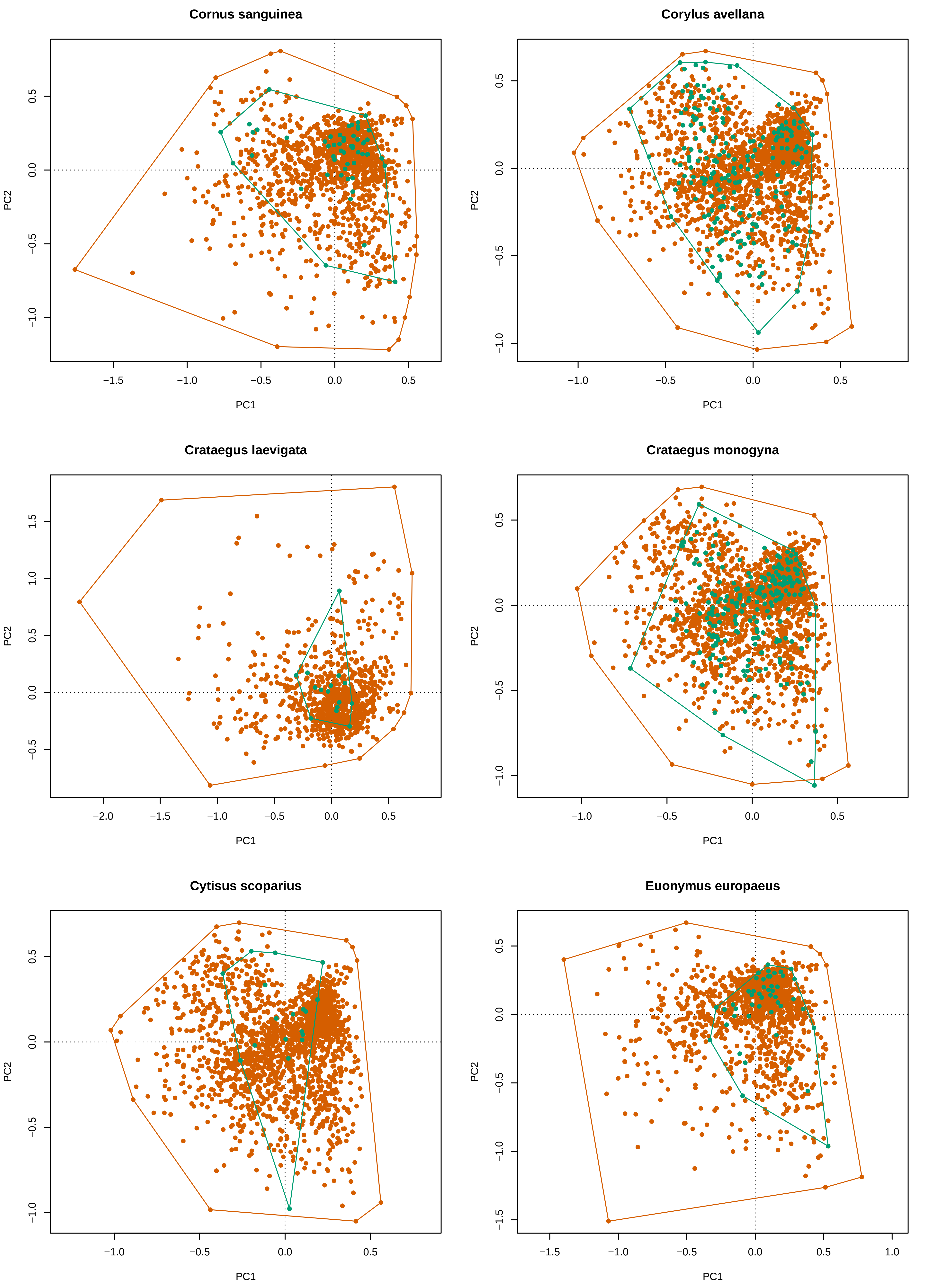
Cornus sanguinea
Corylus avellana
Crataegus laevigata
Crataegus monogyna
Cytisus scoparius
Euonymus europaeus
PC1
PC2

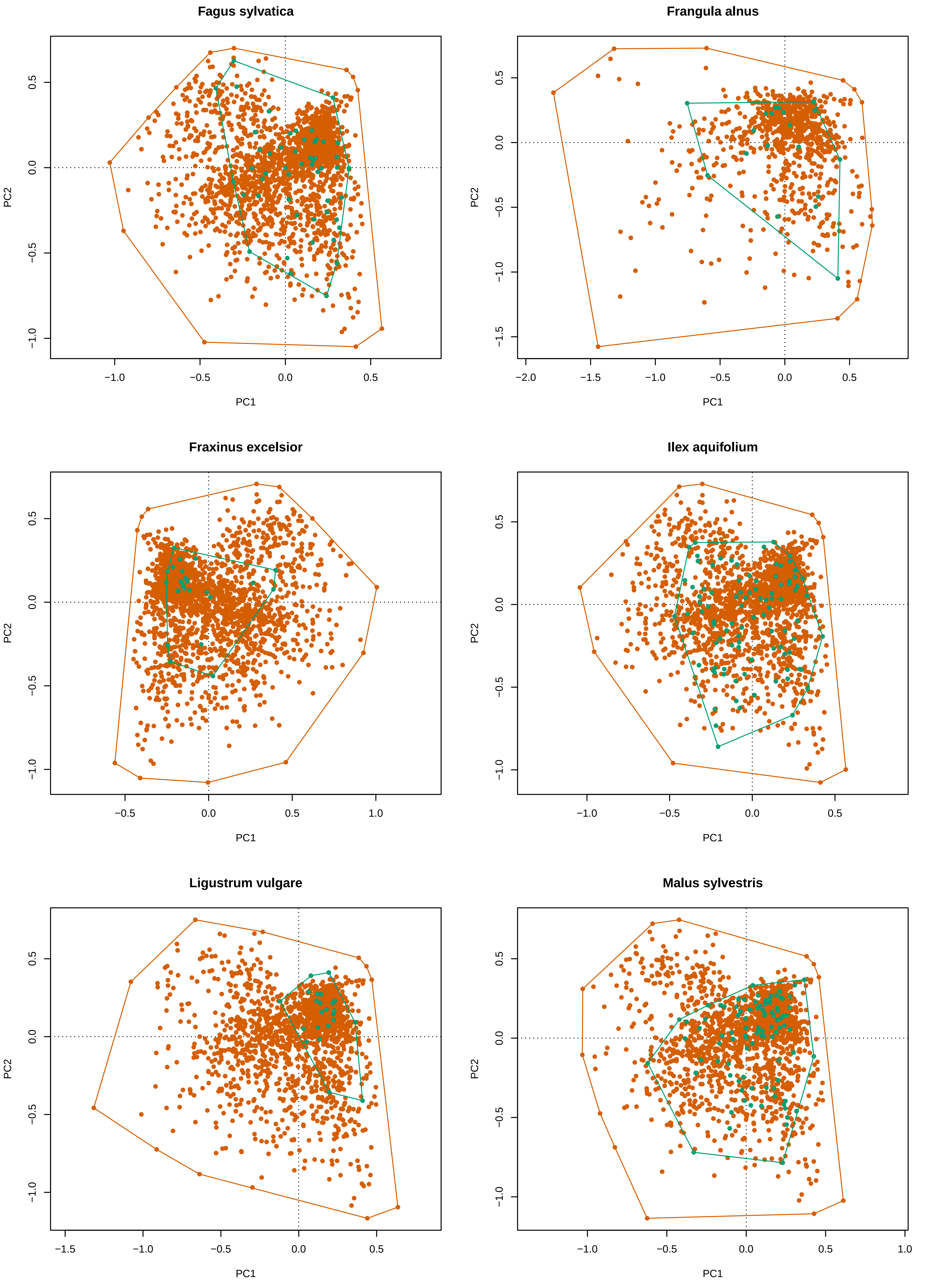
Fagus sylvatica
Frangula alnus
Fraxinus excelsior
Ilex aquifolium
Ligustrum vulgare
Malus sylvestris
PC1
PC2

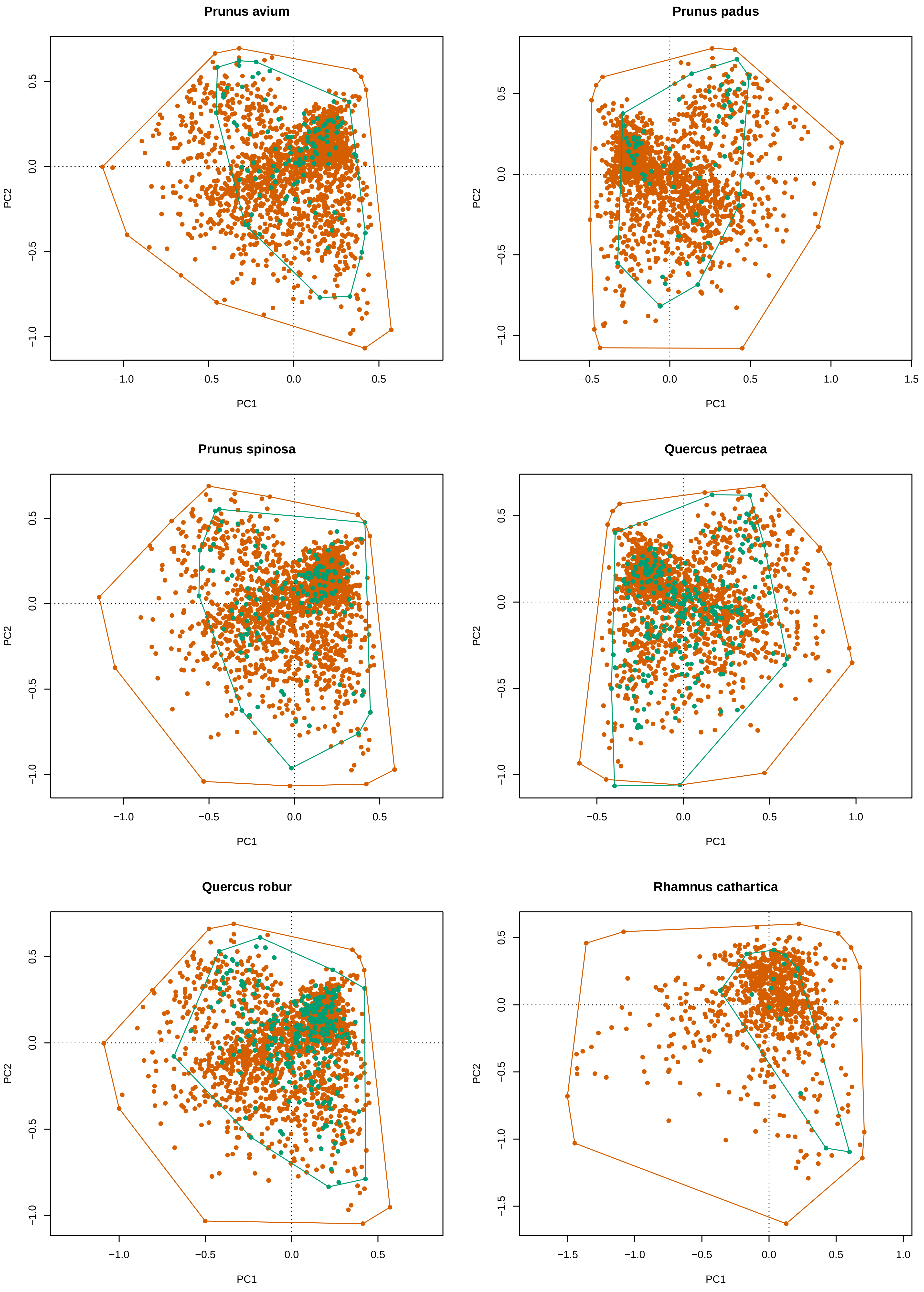
Prunus avium
Prunus padus
Prunus spinosa
Quercus petraea
Quercus robur
Rhamnus cathartica
PC1
PC2
−1.5
−1.0
−0.5
0.0
0.5
1.0
1.5

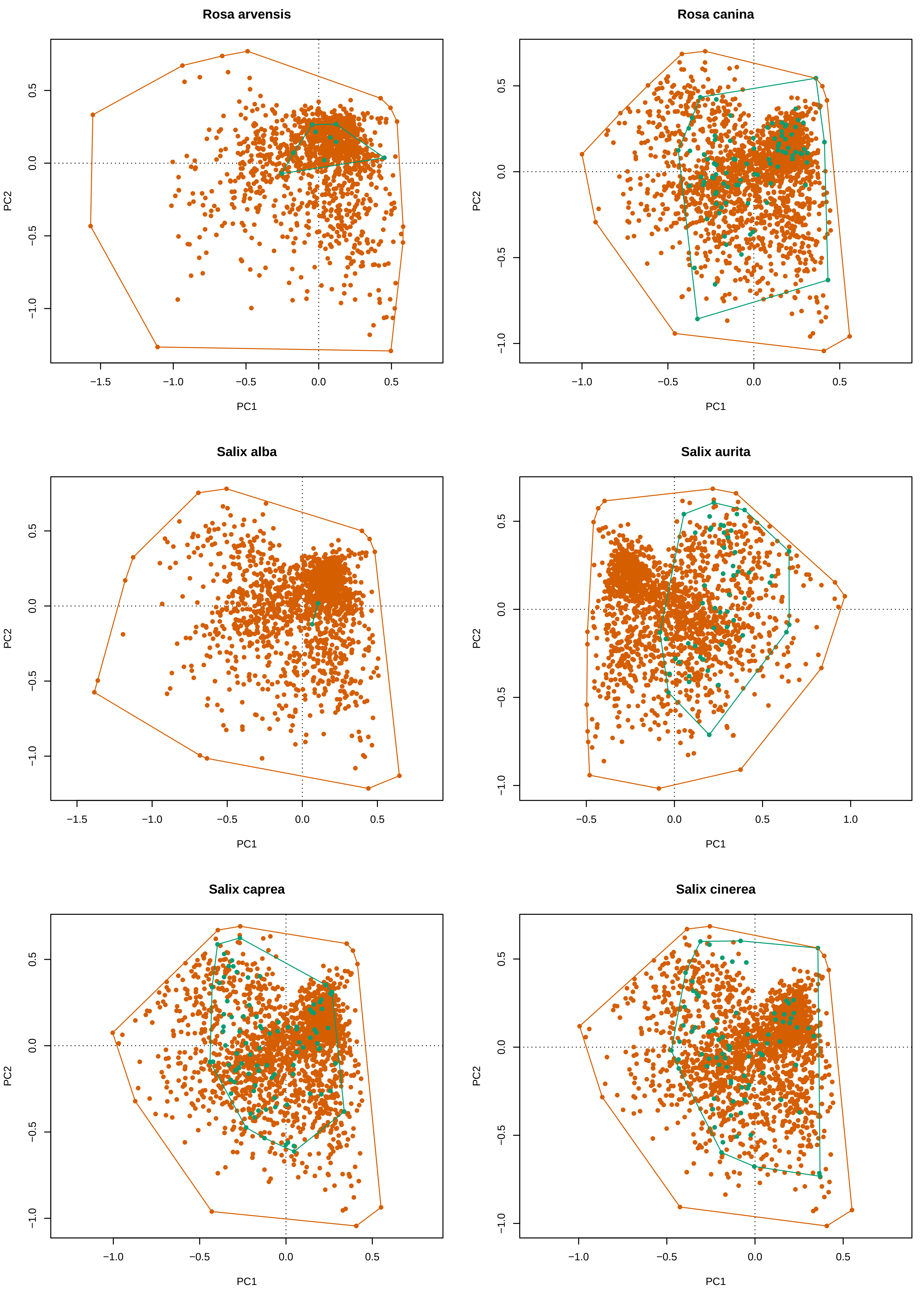

Rosa arvensis
Rosa canina
Salix alba
Salix aurita
Salix caprea
Salix cinerea
PC1
PC2
−1.5
−1.0
−0.5
0.0
0.5
1.0

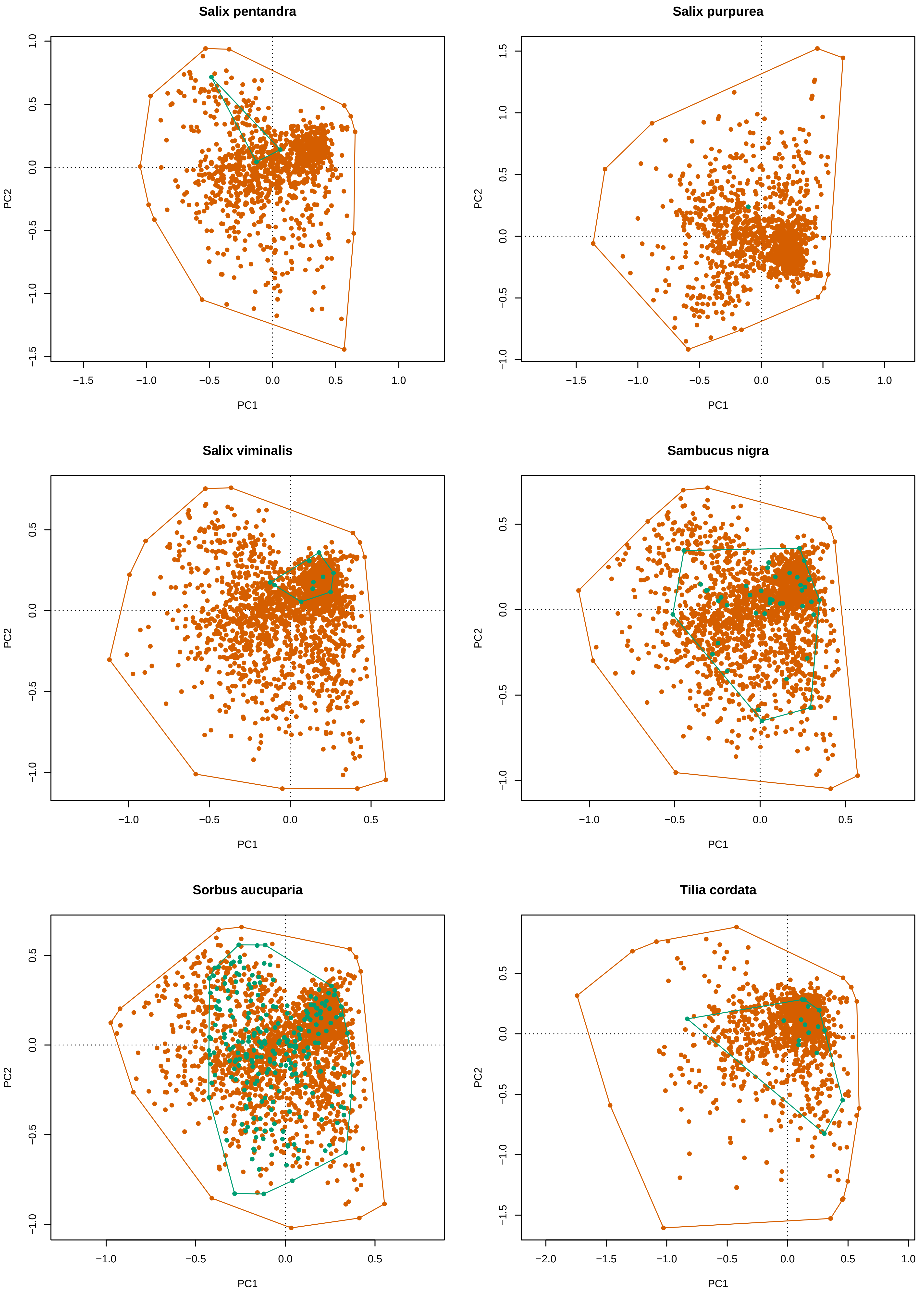
Salix pentandra
Salix purpurea
Salix viminalis
Sambucus nigra
Sorbus aucuparia
Tilia cordata
PC1
PC2

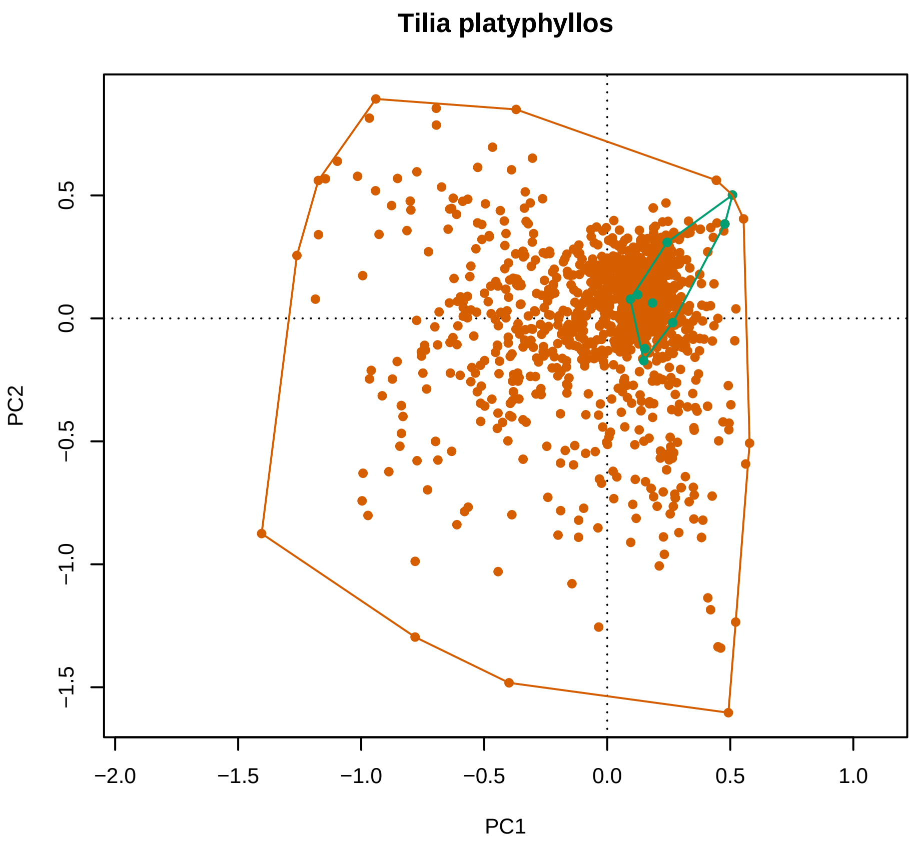
Tilia platyphyllos
PC1
PC2
−2.0 −1.5 −1.0 −0.5 0.0 0.5 1.0
−1.5 −1.0 −0.5 0.0 0.5

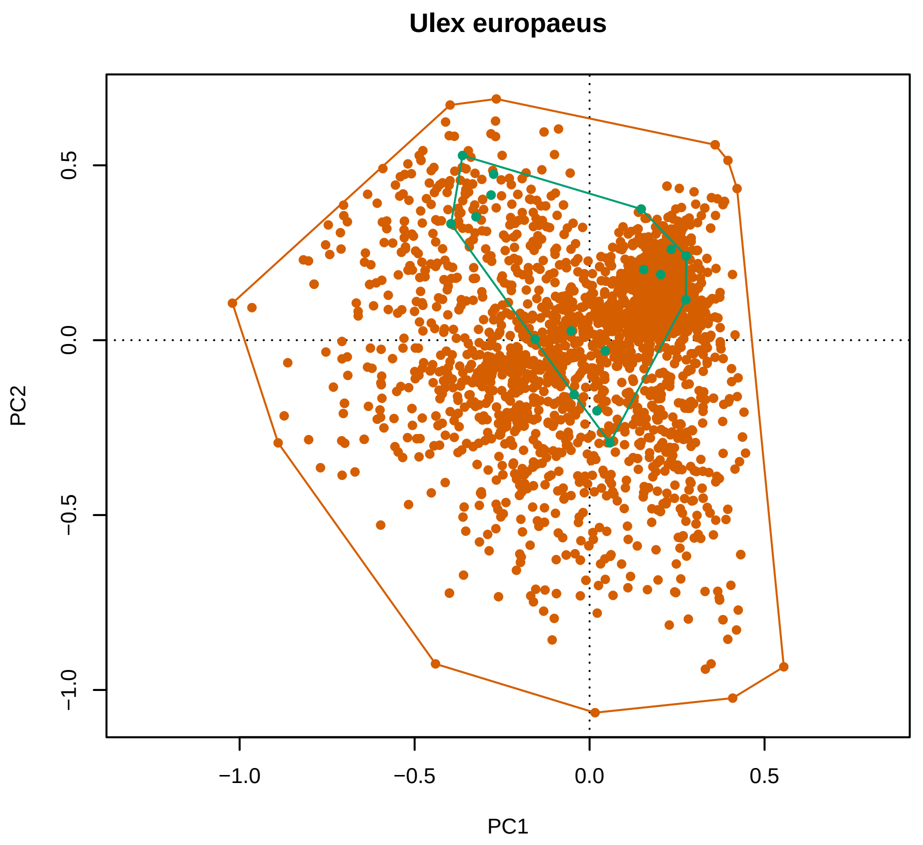
Ulex europaeus
PC1
PC2
−1.0 −0.5 0.0 0.5
−1.0 −0.5 0.0 0.5

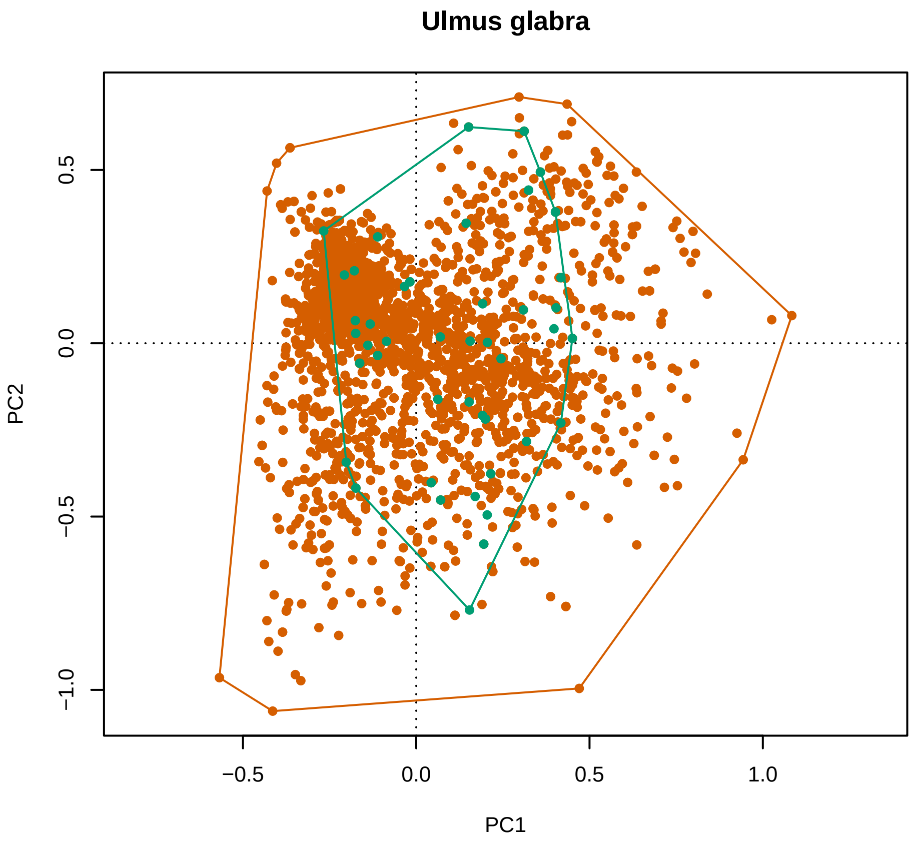
Ulmus glabra
PC1
PC2
−0.5 0.0 0.5 1.0
−1.0 −0.5 0.0 0.5

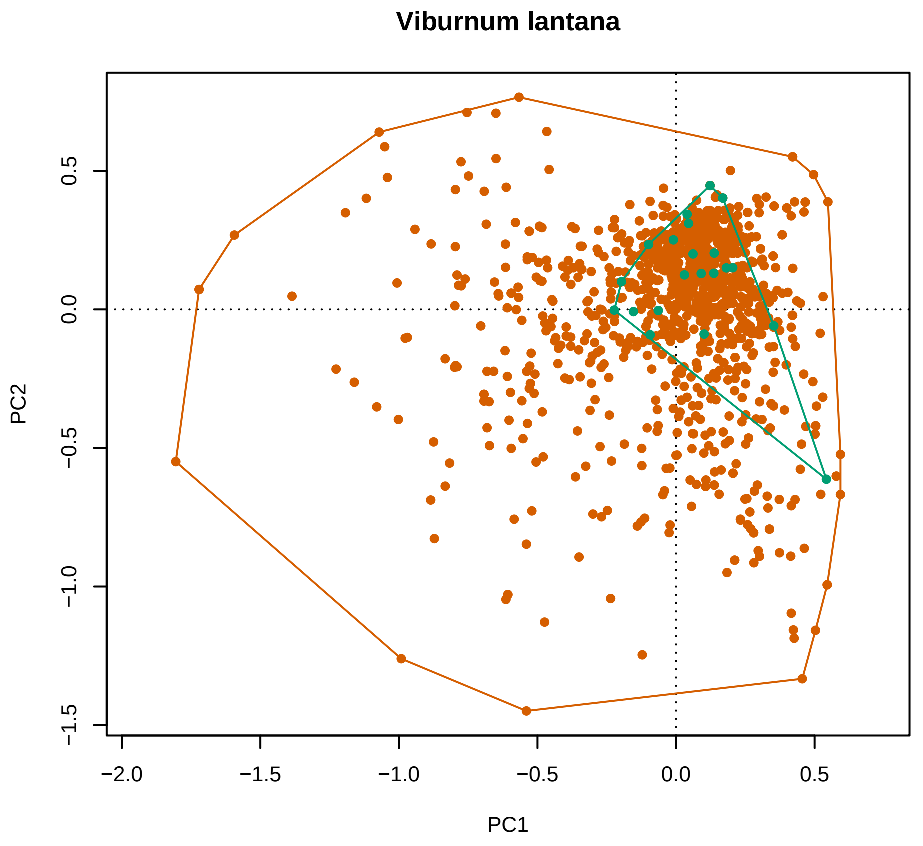
Viburnum lantana
PC1
PC2
−2.0 −1.5 −1.0 −0.5 0.0 0.5
−1.5 −1.0 −0.5 0.0 0.5

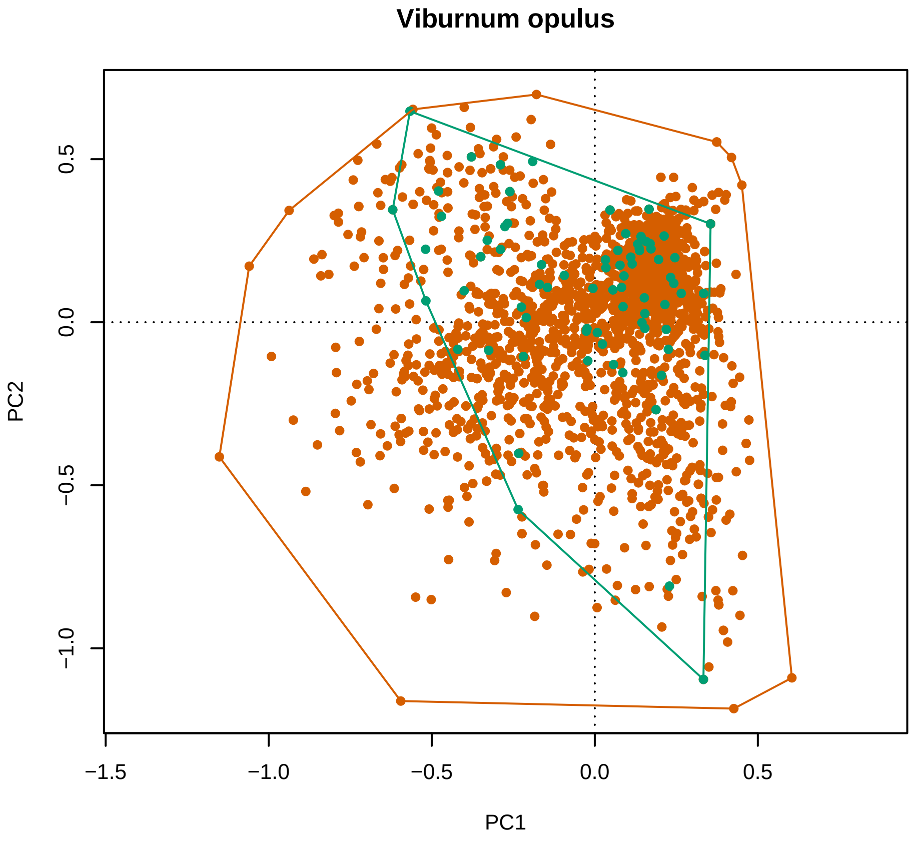
Viburnum opulus
PC1
PC2
−1.5 −1.0 −0.5 0.0 0.5
−1.0 −0.5 0.0 0.5
